# On the Photosensitizing Properties of Aloe-Emodin in Photodynamic Therapy: Insights from the Molecular Modeling

**DOI:** 10.1021/acs.jpcb.5c01117

**Published:** 2025-05-31

**Authors:** Maciej Spiegel

**Affiliations:** Department of Organic Chemistry and Pharmaceutical Technology, Faculty of Pharmacy, 49550 Wroclaw Medical University, Borowska 211A, 50-556 Wroclaw, Poland

## Abstract

The photosensitizing properties of aloe-emodin were investigated under physiological conditions using computational chemistry tools. The neutral and monoanionic species were found to coexist in a 98:2 ratio, with dissociation causing a redshift in the absorption spectrum. Aloe-emodin exhibits high two-photon absorption cross-section values within the therapeutic window and significant transition probabilities, making it an efficient two-photon photosensitizer. Excited-state dynamics analysis revealed a triplet state quantum yield of 0.51 for the neutral species and around 0.88–0.89 for the anionic species, with triplet lifetimes of 26.0 s and 0.66 s, respectively. Both species exhibit similar Type I photoreactivity, but the neutral form more effectively oxidizes biomolecules during Type III photoreactivity. Additionally, the neutral species intercalates into DNA, particularly at the AT–TA site, inducing absorption changes and structural nucleotide rearrangements. The computational results align closely with available experimental data, further confirming their reliability.

## Graphical abstract

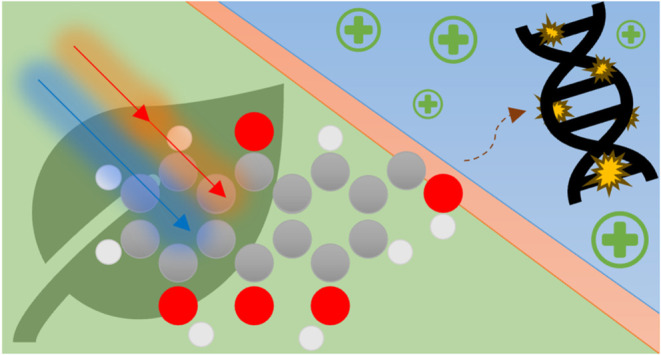

## 1 Introduction

Photosensitizers (Ps) are a class of photoactive dyes that undergo a series of photoreactions when exposed to photons of specific energies. Their remarkable versatility makes them pivotal in diverse applications, including photocatalysis,^1^ solar energy conversion,^2^ and medicine. Among these applications, photodynamic therapy (PDT) stands out as a clinically approved, noninvasive treatment modality for malignancies,^3^ skin diseases,^4^ and microbial infections.^5^ Often referred to as a “molecular scalpel”, PDT offers precise and localized action at the treatment site. Photosensitizers operate through distinct mechanisms, all originating from excitation to a higher-lying singlet state—dependent on the absorbed wavelength—followed by radiative and nonradiative processes such as internal conversion to lower-lying singlet states (S) or intersystem crossing (ISC) to energetically proximate triplet states (T). ISC is crucial for phototoxicity because it generates the initial triplet state that subsequently initiates cytotoxic photoreactions.

Two primary mechanisms—Type I and Type II^6,7^ —are generally recognized, with their prevalence depending on the oxidative environment within cells. In Type I reactions, reactive oxygen species (e.g., hydroxyl radicals) are generated via electron transfer processes. In Type II mechanism, energy transfer from the triplet state of the photosensitizer to molecular ground-state triplet oxygen (^3^O_2_) produces highly cytotoxic singlet oxygen (^1^O_2_).^8^ More recently, a Type III mechanism has been proposed, which involves direct interactions with biomolecules, leading to their degradation through electron-mediated processes. This pathway is particularly advantageous in hypoxic environments, such as those found in aggressive tumors.^9,10^ Regardless of the mechanism, the cumulative cellular damage eventually results in cell death and the destruction of the target tissue.^11^

PDT has gained widespread acceptance due to its high therapeutic efficacy and reduced side effects compared to conventional treatment approaches. It effectively addresses challenges such as cancer cell resistance to platinum-based chemotherapeutic agents^12^ or bacterial resistance to antibiotics.^13^ Furthermore, approved photosensitizers generally exhibit fewer adverse effects because they selectively accumulate at the treatment site and display temporally controlled activity due to their photochemical decay properties.^14^

An ideal photosensitizer should exhibit several key properties: (a) no dark toxicity or intermolecular aggregation that could impair photoactivity; (b) solubility in aqueous media; (c) redox stability under physiological conditions; (d) a T_1_ state with a sufficiently long lifetime to participate effectively in photoreactions; and (e) strong absorption within the phototherapeutic window, ensuring optimal tissue penetration while minimizing off-target absorption.^9,15^ These attributes are critical for maximizing therapeutic efficacy against cancer while minimizing side effects. In contrast, antimicrobial photodynamic therapy—which has emerged as an effective means to eradicate resistant microbes on superficial surfaces—offers greater flexibility in photosensitizer design.^16^

Numerous photosensitizers have been developed, many based on rare metals such as Ru­(II), Re­(I), Os­(II), or Ir­(III) coordinated with *i.e.*, porphyrinoid or phenanthroline scaffolds.^17^ Although these compounds meet stringent performance criteria and exhibit potent photoactivity, their high production costs impose a financial burden and raise environmental concerns due to resource-intensive and potentially harmful manufacturing processes. Moreover, many of these compounds do not possess ideal photodynamic properties. As an alternative approach to overcome the limitations of one-photon absorption (OPA), a two-photon absorption (TPA) strategy has been proposed. TPA enables near-infrared absorption, allowing treatment of deeper tissues with higher spatial resolution due to localized absorption at high photon-density sites. This localized absorption decreases background noise and reduces photodamage to surrounding tissues.^18,19^

These challenges and opportunities have spurred interest in exploring phytochemicals for their photosensitizing properties.^20,21^ Notable examples include the use of psoralens in PUVA therapy for skin diseases^22^ and the development of mitochondria-targeting coumarin-based fluorophores for cancer treatment.^23^ Another promising group of compounds is the 9,10-anthraquinones, which exhibit significant therapeutic potential in both anticancer^24–26^ and antimicrobial photodynamic therapies.^27–29^

A key representative of the 9,10-anthraquinone group is aloe-emodin (Figure 1), a compound readily derived from the Aloe vera plant. Aloe-emodin has demonstrated notable therapeutic potential in oncology, including the inhibition of human oral squamous cell carcinoma^30^ and basal cell carcinoma.^31^ Beyond its antitumor activity, aloe-emodin exhibits antimicrobial efficacy against pathogens such as Staphylococcus aureus,^32^ Acinetobacter baumannii,^33^ Pseudomonas aeruginosa,^34^ Trichophyton rubrum,^35^ and drug-resistant Candida albicans strains.^36^ Additionally, it has been successfully applied in the treatment of superficial diseases.^35,37^ These effects are attributed to its photodynamic properties, which arise from its absorption in the blue-light region.^37^ Although this lies outside the typical therapeutic window, it is particularly advantageous for targeting superficial tissues, as light in the 400–450 nm range penetrates to depths of approximately 300–400 μm, thereby minimizing damage to deeper healthy tissues.^38,38^ Aloe-emodin holds great promise as a photosensitizer, notably because it does not exhibit dark toxicity.^39,40^ However, robust evidence detailing the molecular mechanisms underlying its photodynamic activity remains scarce.^37,39–41^ Experimental studies are limited, and a deeper understanding of how this anthraquinone interacts with biomolecules at the cellular level during PDT is essential for optimizing its therapeutic potential. To address this knowledge gap, the present study provides a comprehensive investigation of these mechanisms.

**Figure 1:**
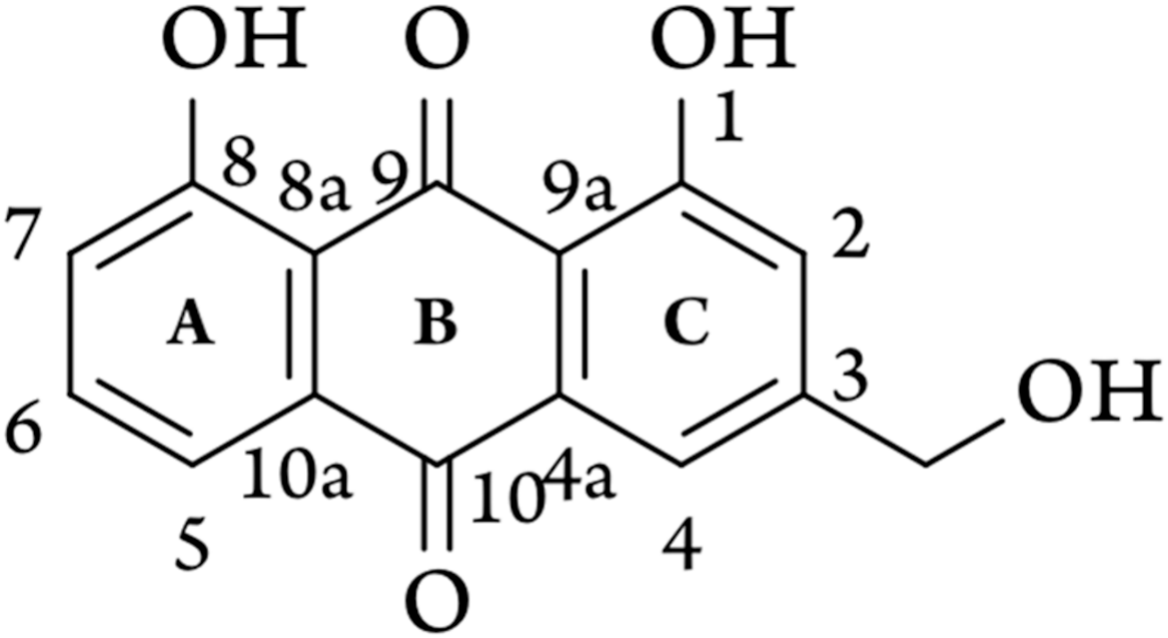
Reference 2D structure of aloe–emodin with IUPAC numbering.

In this paper,^42^ density functional theory (DFT) and its time-dependent counterpart (TD–DFT) are employed to examine the geometric and photophysical properties of the singlet and triplet states of aloe-emodin species present at physiological pH. The systematic study encompasses one- and two-photon absorption properties, excited-state dynamics (including process rates and triplet lifetimes), thermochemical and kinetic analyses of the feasibility of Type I, II, and III photoactivity, and the compound’s capacity to intercalate into DNA and enhance photooxidative damage. This research is expected to stimulate further investigations into this class of compounds, potentially leading to the development of novel photosensitizers.

## 2 Computational Methods

The methodology outlined below pertains to the evaluation of aloe-emodin’s photoactivity. Detailed information regarding structure generation, acid–base equilibria, and the TD–DFT benchmarks for one-photon absorption and two-photon absorption are provided in the Supporting Information to enhance the clarity of the main text.

Photophysical properties were computed using the *Orca* (v. 6.0)^43^ software package. Solvent effects were included using the Universal Solvation Model Based on Solute Electron Density (SMD).^44^ Throughout the calculations, tight SCF convergence criteria, D4 dispersion corrections,^45^ and a machine-learning-optimized, high-density integration grid were employed to ensure enhanced accuracy.^46^ Computational efficiency was further improved by using the “chain-of-spheres” algorithm for Hartree–Fock exchange^46,47^ and the “resolution-of-the-identity” approximation for the Coulomb matrix.^48,49^

The basis set used was ma–def2–TZVP,^50^ coupled with an auxiliary Coulomb-fitting basis set.^51^ This selection was driven by the necessity to obtain accurate energetics, particularly due to the strong impact of diffuse functions on electron affinity^50^—critical for accurately determining the propensity for Type I and III activity. A benchmark, as presented in the Supporting Information, confirmed that O3LYP,^52^ when addressing triplet instabilities using the Tamm–Dancoff approximation,^53^ provides excellent accuracy and reliability.

Rate constants for intersystem crossing (*k*_ISC_) between the *n* ^th^ singlet excited state (S*_n_*) and the *m* ^th^ triplet state (T*_m_*), as well as for fluorescence (*k*_F_), internal conversion (*k*_IC_) from S_1_ to the ground state, and phosphorescence (*k*_P_) from T_1_, were calculated analytically using Fermi’s Golden Rule-like equations,^54,55^ as implemented in *Orca*. To account for vibronic transitions, rotational coupling, and vibronic contributions to formally forbidden transitions, the adiabatic Hessian approach, Duschinsky rotation, and Herzberg–Teller corrections were applied, respectively.

The two-photon absorption profile of aloe–emodin was assessed using quadratic response theory,^56^ available in the Dalton (v2020.0) code.^57^ Based on benchmark results, the B3LYP/aug-cc-pVDZ level of theory provided the most reliable TPA predictions and was therefore employed for the calculations. The two-photon cross-section (σ_TPA_) was derived from the rotationally averaged TPA strength (⟨δ_TPA_⟩), following eq 1

1 $$\sigma_{\mathrm{TPA}}=\frac{N\pi^{3}\alpha a_{0}^{5}\omega^{2}}{c}\left\langle \delta_{\mathrm{TPA}}\right\rangle g\left(2\omega ,\omega_{0},\Gamma \right)$$

where *N* is an integer, α is the fine-structure constant, *a*_0_ is the Bohr radius, ω is the photon energy in atomic units, *c* is the speed of light in vacuum, and *g*(2ω,ω_0_,Γ) is the line shape function describing spectral broadening effects. The obtained σ_TPA_ was corrected in two ways^58,59^

(1) Degeneracy—The default Dalton setting assumes an experimental setup with two laser sources, for which *N* = 8. However, in the commonly applied single-beam experiment, both absorbed photons are degenerate, resulting in *N* = 4. Therefore, σ_TPA_ was halved.
(2) Solvent effects—A Lorentzian line shape function was refined to a better-fitting Gaussian one by multiplying σ_TPA_ by √(ln(2)­π)(≈1.4757).

Consequently, the final σ_TPA_ is determined as follows (eq 2)­

2 $$\sigma_{\mathrm{TPA}}=\frac{1.4757\sigma_{\mathrm{TPA}}}{2}$$

Henceforth, this formulation should be understood whenever σ_TPA_ is mentioned in the text.

The reaction rates for Type I and Type III phototoxicity were determined using conventional transition state theory, as expressed by eq 3

3 $$k=\frac{k_{b}T}{h}e^{-\left(\Delta G^{\neq }/\mathrm{RT}\right)}$$

where *k*_b_, *h*, and *R* are the Boltzmann constant, Planck constant, and ideal gas constant, respectively; *T* is the temperature (set at 298.15 K); and Δ*G* ^≠^ is the activation energy. Given the electron-transfer character of the reactions, the Δ*G* ^≠^ values were calculated within the framework of Marcus theory using eq 4

4 $$\Delta G^{\neq }=\frac{\lambda }{4}{\left(1+\frac{\Delta G}{\lambda }\right)}^{2}$$

where Δ*G* is the adiabatic Gibbs free energy of the reaction, and λ is the reorganization energy. The λ was determined using a previously tested approximation,^60,61^ given in eq 5

5 λ≈ΔE−ΔG

where Δ*E* represents the vertical energy difference—defined as the energy difference between the products and the reactants at the reactant’s equilibrium geometry, without geometric relaxation of the product.

Molecular dynamics (MD) simulations were carried out as follows: ground-state geometries were assigned RESP charges^62,63^ using the *antechamber* program from AmberTools.^64^ Any missing parameters were generated using *parmchk2* with the GAFF2 force field.^65^ The B–DNA structure (PDB ID: 1BNA^66^) was prepared using *LEaP* and *CPPTRAJ* program,^67^ and aloe-emodin was manually intercalated into three base pair steps (AT–TA, CG–GC, and TC–AG) to assess binding selectivity. This procedure, supported by previous studies,^68–70^ provides an efficient and cost-effective methodology to examine intercalator:DNA interactions.

The resulting systems were solvated in a 9.0 Å octahedral OPC water box, and charge neutralization was achieved by incorporating 11 Mg^2+^ ions modeled with TIP4P-Ew,^71^ which are particularly effective in stabilizing nucleic acids.^72,73^ To mimic physiological conditions, 12 Na^+^ and 12 Cl^–^ ions^74,75^ were added—calculated using the *STLCAP* method^76^—to achieve a final salt concentration of 0.150 M. Additional monovalent ions introduced as needed to maintain neutrality. Hydrogen mass repartitioning was performed with *PARMED* ^77^ to enable longer simulation time steps. Simulations were then conducted following an established protocol,^78^ and RMSD analysis of a 100 ns production run was performed using *CPPTRAJ*.

Thermochemical aspects of binding were evaluated using Molecular Mechanics/Poisson–Boltzmann Surface Area (MMPBSA) calculations.^79^ Topologies were generated with the *pre-MMPBSA.py* script and analyzed using *MMPBSA.py* ^80^ from the *Amber24* package. Default parameters were used, with the ionic strength set to 0.150 M to match the explicit simulation conditions. For systems with favorable (i.e., negative) binding energies, noncovalent interaction (NCI) analysis^81^ was performed using the reduced density gradient (RDG) framework^82^ available in *Multiwfn* (v. 3.8).^83^ Representative geometries were selected through *k*-means clustering, and “minimal” systems^68–70^ were constructed by retaining only the intercalator, the two intercalated base pairs, and their immediate neighbors. These minimal systems were reoptimized using the *r* ^2^SCAN-3c composite method,^84^ while constraining the coordinates of the terminal nucleotides to avoid bias due to their free motion. Implicit solvation was applied using the SMD water model, and their absorption spectra were computed with the simplified Time-Dependent Approximation (sTDA)^85^ at the same level of theory, with ma-def2-TZVP replaced by its not-augmented version^86^ for improved convergence.

## 3 Results and Discussion

### 3.1 Acid–Base Equilibria

Aromatic phenolic groups are mildly acidic, and compounds containing these groups can exist in different protonation states depending on the pH. The methodology applied in this study allowed for the determination of p*K*_a_ values and identification of the preferred deprotonation pathways, thereby facilitating the calculation of molar fractions (*m*_f_) under the investigated conditions (see Figures 2 and S2).

**Figure 2:**
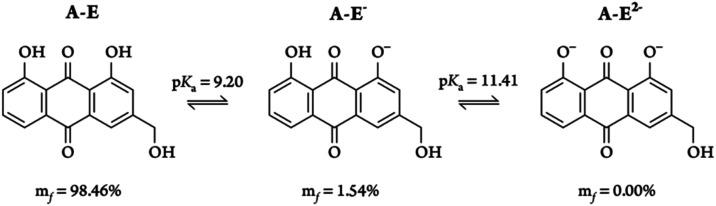
Deprotonation pathways, dissociation constants, and molar fractions of aloe-emodin at physiological pH.

The deprotonation process follows a sequential pathway, beginning with the dissociation of the hydroxyl group at C_1_, as previously suggested,^40^ followed by the dissociation of the hydroxyl group at C_8_. The energetics of these steps yielded p*K*_a_ values of 9.20 and 11.41 for the first and second deprotonation events, respectively. The value obtained for the first dissociation constant is in excellent agreement with the experimental value (9.4 ± 0.2).^40^ Although no experimental value for the second p*K*_a_ is currently available, the reliability of the chosen methodology (mean absolute error = 0.3 kcal mol^–1^)^87^ suggests that significant deviations from the actual value are unlikely.

Under physiological conditions, the neutral (**A–E**) form and the anionic (**A–E** ^–^) form are present at molar fractions of 98.46% and 1.54%, respectively, while the dianionic form is negligible. These results indicate that phototoxicity in aqueous media should be primarily associated with the neutral species, with the anionic form playing a minimal role. Nevertheless, both species were considered in subsequent analyses to provide a broader perspective.

### 3.2 One-Photon Absorption

Figure 3 displays the simulated OPA spectra of **A–E** and **A–E** ^–^ in water, based on vertical excitation energies. For **A–E**, the first bright state is located at 454.9 nm, whereas for **A–E** ^–^, it appears at 532.9 nm. Upon deprotonation, a new absorption band emerges in the range of approximately 325–375 nm, along with a bathochromic shift of the first bright state—features that are consistent with experimental observations.^40,88^ Notably, the most intense absorption peak for **A–E** ^–^ corresponds to the S_2_ excitation; the S_1_ state, located at 581.7 nm, remains nearly invisible due to its extremely low oscillator strength (*f* ≈ 1.414 × 10^–5^). To rationalize these spectral characteristics, an analysis of the frontier molecular orbitals was conducted and is presented later in the text. The absorption spectrum for the dianionic form (see Figure S3) reveals that the S_1_ state also remains dark at 694.4 nm, with only the S_2_ state showing appreciable intensity at 583.8 nm. This findings indicate a reduction in excitation energy at higher pH and a concomitant reorganization of the molecular orbitals involved in the transitions—albeit at the cost of diminished excitability, as reflected in the lower spectral intensity of the S_1_ state.

**Figure 3:**
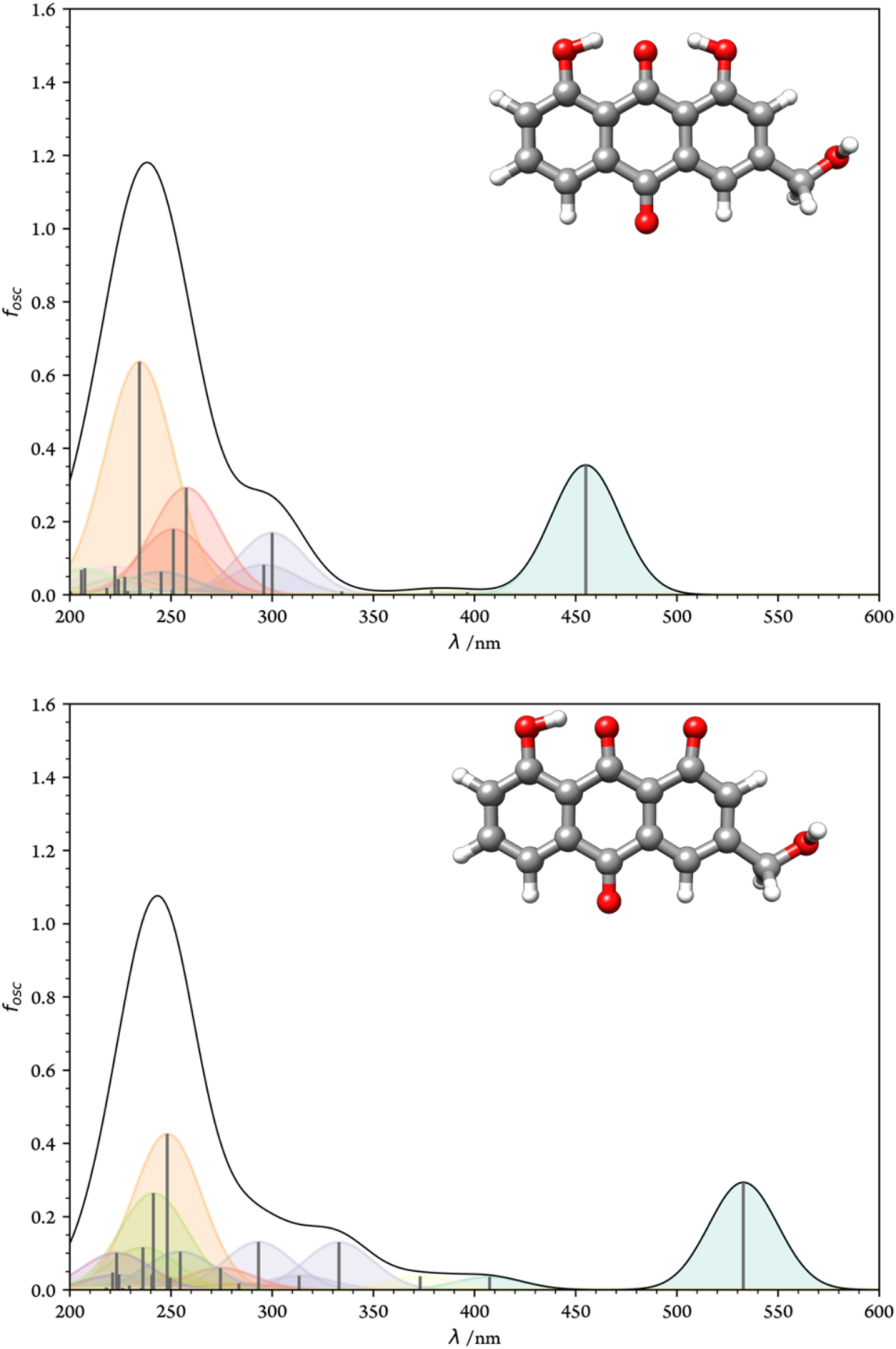
One–photon absorption spectra of **A–E** (top) and **A–E** ^–^ (bottom) in water.

The HOMO–LUMO gaps (*E*_H–L_) were determined to be 2.78 eV for **A–E** and 2.23 eV for **A–E** ^–^. The inaccessible S_1_ transition is associated with an energy gap of 2.62 eV, slightly lower than that of the nondissociated species. For the dianion, the HOMO–LUMO gap further decreases to 2.16 eV, indicating that deprotonation leads to a narrowing of the energy gap.

In the visible S_1_ state of **A–E** (*f* = 0.354), the excitation is predominantly composed of an H → L electronic transition (94.73%). In contrast, for **A–E** ^–^, the first excited state originates almost entirely from the H–1 → L transition (99.33%), while the S_2_ state is primarily composed of the H → L transition (92.37%) (see Table S2).

The significant contributions of individual molecular orbitals to these excitations suggest that the Natural Transition Orbitals (NTOs) closely resemble the corresponding molecular orbitals. As illustrated in Figure 4, both the S_0_ → S_1_ transition of **A–E** and the S_0_ → S_2_ transition of **A–E** ^–^ involve electron-density shifts from aromatic π-bonds to their corresponding antibonding orbitals. This analysis supports the notion that the previously discussed dark excitation arises from symmetry-forbidden electron transitions, resulting in near-zero dipole moments and minimal oscillator strengths—rendering them nearly invisible in the absorption spectrum.

**Figure 4:**
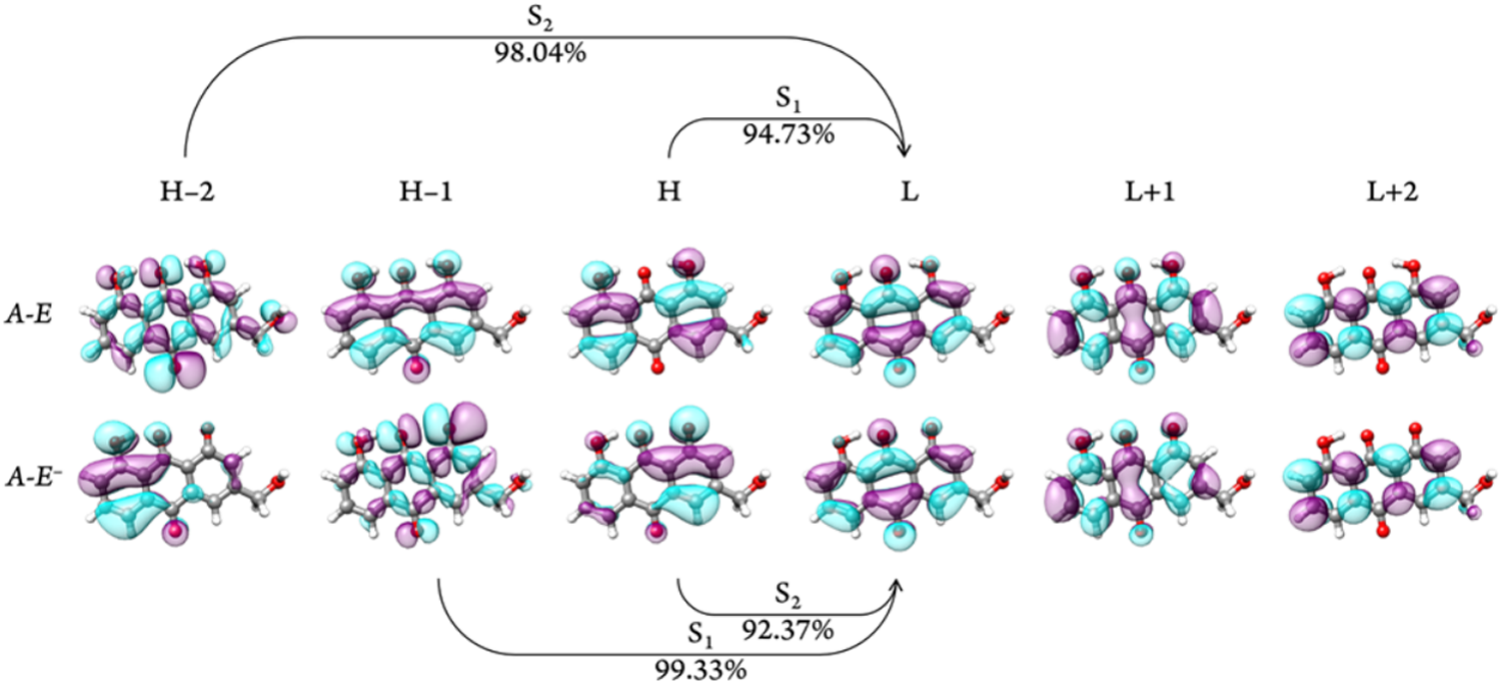
Molecular orbitals involved in the main absorption transitions of **A–E** and **A–E^–^**.

### 3.3 Two-Photon Absorption Profile

It is well established that strongly conjugated aromatic systems can serve as effective photosensitizers for TPA applications.^89^ The extended π-electron cloud of both **A–E** and **A–E** ^–^ makes these compounds promising candidates for such purpose. The effective photon wavelength (λ_TPA_, in nm, calculated as half the excitation energy) and the two-photon absorption cross section (σ_TPA_, in GM) were computed for the first 10 excited states. The resulting TPA spectra under linearly polarized lights, along with the corresponding data points, are presented in Figure 5 and Table S4.

**Figure 5:**
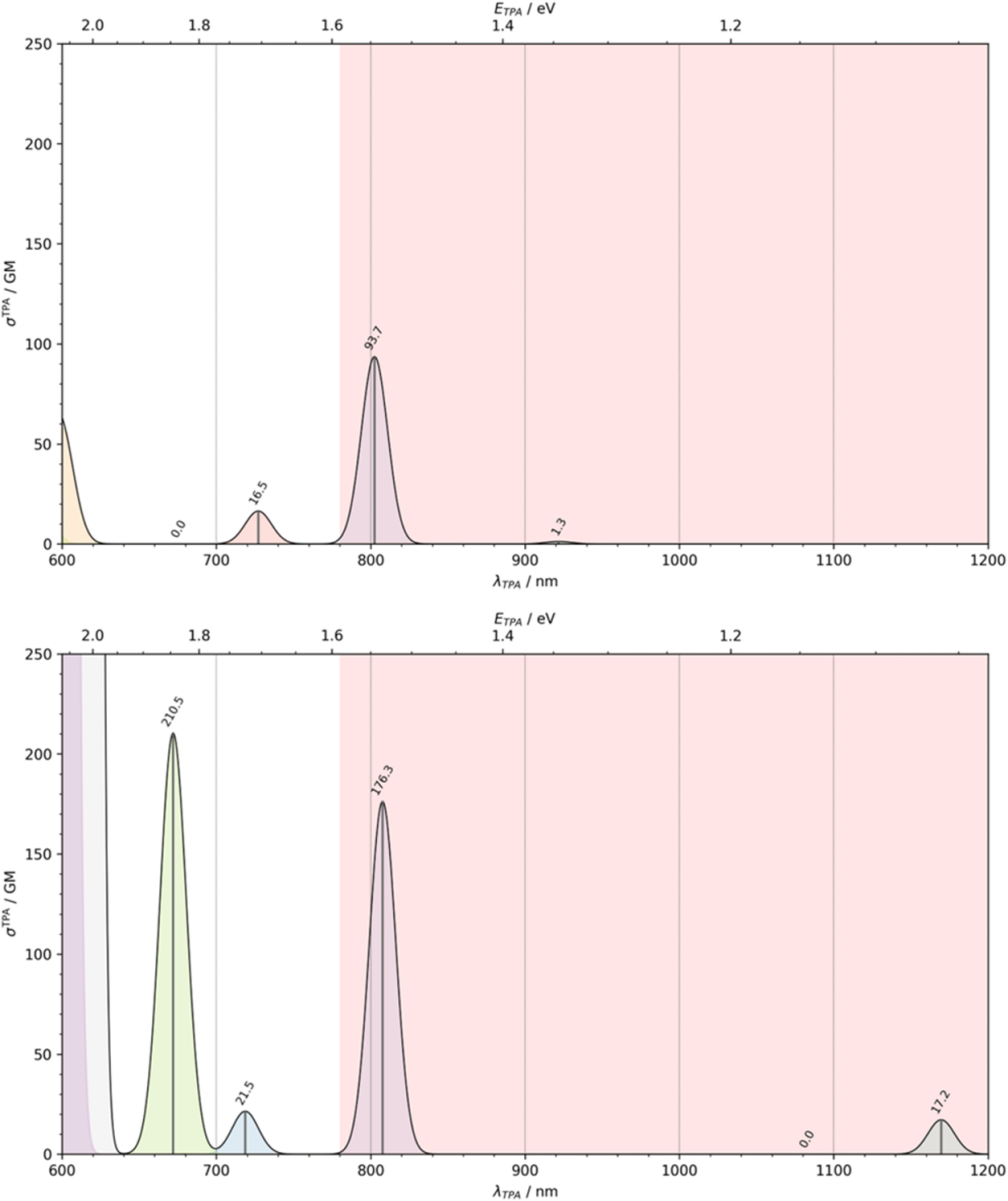
Two-photon absorption spectra of **A–E** (top) and **A–E** ^–^ (bottom) in water under linearly polarized laser beam. The red-shaded area denotes the therapeutic window.

According to the results, **A–E** exhibits two TPA peaks within the near-infrared region, corresponding to the S_0_ → S_1_ and S_0_ → S_3_ transitions. The former transition displays a negligible cross-section of only 1.3 GM, whereas the latter, occurring at 802.5 nm, exhibits a significantly higher σ_TPA_ of approximately 93.7 GM. In contrast, **A–E** ^–^ shows markedly enhanced TPA activity. The S_0_ → S_1_ transition becomes more accessible, occurring at a substantially longer wavelength (1169.7 nm), and exhibits a modestly increased σ_TPA_ of 17.1 GM. Notably, the S_0_ → S_3_ transition retains nearly the same λ_TPA_ as in the neutral form but shows a a dramatic enhancement in cross section, reaching 176.3 GM—almost a twofold increase. This trend continues for higher-energy transitions, particularly those around 672.0 nm and near 600 nm. These findings lead to a clear conclusion: deprotonation significantly enhances transition probabilities, as reflected in the increased σ_TPA_ values across the spectrum.

Rather than focusing on absolute agreement with experimental σ_TPA_ values, a comparative approach is more appropriate, as discussed in the Supporting Information. Using emodin—a known and experimentally validated two-photon photosensitizer for antineoplastic therapy^90^—as a reference, aloe-emodin demonstrates even greater activity, especially in its anionic form. This suggests that aloe-emodin may serve as a more effective photosensitizer for similar anticancer applications. Other related examples support this perspective. *Trans*-stilbenes, for instance, exhibit a wide range of corrected σ_TPA_ values (from 8.9 GM to 1431.4 GM), depending on their substitution pattern.^91,92^ In comparison, coumarins typically display lower σ_TPA_ values than those calculated for aloe-emodin.^59^

Dyes suitable for PDT are known to be highly sensitivite to molecular structure alternation—particularly the nature of terminal groups and the extent of π-conjugation, which can strongly influence *via* push–pull effect.^18^ For example, the alkaloid sanguinarine exhibits a corrected σ_TPA_ of 6550 GM at 715 nm,^93^ likely due to its extensive conjugation and strong electron-donating and -withdrawing substituents. Although not directly relevant to TPA, recent structural modifications of the coumarins have yielded outstanding OPA photosensitizers.^23,94^ Given the promising TPA properties of anthraquinones shown here, structural modifications could further enhance their two-photon cross sections and expand their utility in two-photon photodynamic therapy.

In summary, the ability of aloe-emodin to absorb two photons within the therapeutic window provides a distinct advantage over conventional OPA limitations, enabling deeper tissue penetration for clinical applications. Moreover, its enhanced σ_TPA_ in the 600–800 nm range makes it especially suitable for treating superficial conditions such as melanoma, mucosal cancers, or for use in antimicrobial photodynamic therapy.

### 3.4 Excited State Dynamics and Type II Phototoxicity

Upon photoexcitation, a molecule absorbs energy and transitions to an excited singlet state. This process triggers rapid, nonradiative vibrational relaxation, bringing the molecule to the vibrational ground state of the excited electronic level. From this point, the molecule may follow one of two primary decay pathways. It can either undergo intersystem crossing—a process facilitated by spin–orbit coupling—leading to population of an energetically accessible triplet state, or it may return to a lower singlet state through internal conversion, a nonradiative process driven by the coupling of vibrational modes with similar energies between the involved electronic states. Regardless of the pathway taken, Kasha’s rule^95^ applies: fluorescence arises from the vibrational ground state of the first excited singlet state, while phosphorescence originates from the vibrational ground state of first triplet state, regardless of the initially excited state.

To visualize the accessible decay routes and the excited-state dynamics, Jablonski diagrams (Figure 6) were generated using a customized version of the *PyEnergyDiagrams.py* ^96^ script. These diagrams are based on adiabatic excitation energies. In the case of the anionic form, additional decay pathways originating from the S_2_ state were also examined.

**Figure 6:**
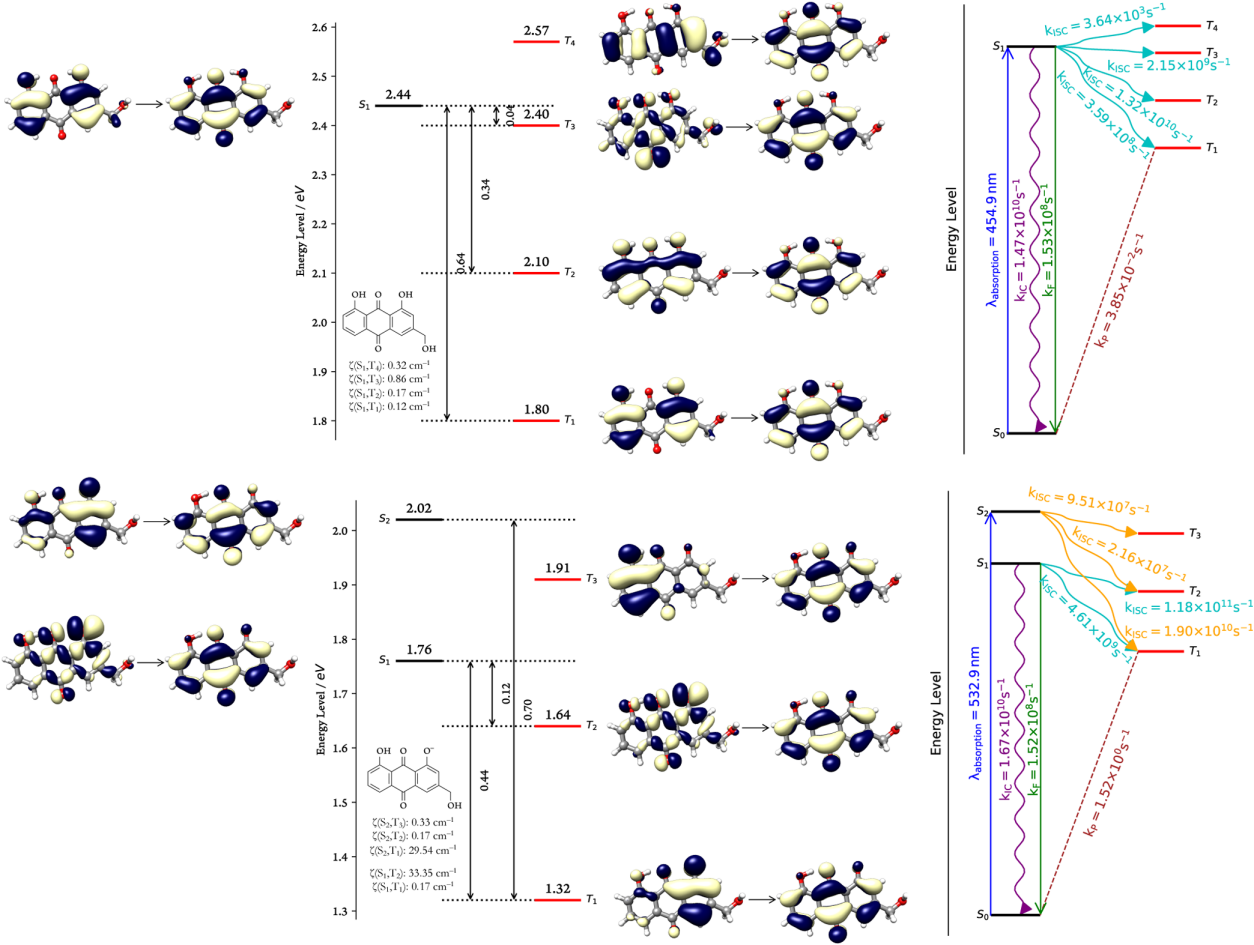
Jablonski diagrams of singlet and triplet excited states for **A–E** (top) and **A–E–** (bottom), with energies referenced to their respective S_0_ states and annotated with spin–orbit coupling values (ζ).

A key observation is that the energy gap between the T_1_ and S_0_ states exceeds 0.98 eV for both **A–E** and **A–E** ^–^. This threshold is critical because it represents the minimum energy required to convert ^3^O_2_(^3^∑_g_^–^) into ^1^O_2_(^1^Δ_g_) through energy transfer from the photosensitizer’s triplet state. This process is a defining step in Type II photoreactivity,^97^ presented in eq 6

6 Ps(T1)+O23(∑g−3)→Ps(S0)+O21(1Δg)

Examining further, although only two triplet states lie below the S_1_ of **A–E** ^–^, they are energetically closer to it than in **A–E**. For the latter, the energy differences are approximately 0.64 eV (S_1_ → T_1_) and 0.34 eV (S_1_ → T_2_), with the S_1_ → T_3_ gap estimated at about 0.05 eV. Given that ISC is highly sensitive to even small energy differences—as described by eq 7—these variations are expected to significantly affect the rate of the process. Notably, the computed T_1_ energy for **A–E** deviates by ca. 0.17 eV from the experimental value,^40^ supporting the reliability of the computational approach.

7 $${k\left(\omega \right)}_{S_{n}T_{m}}=\frac{2\pi }{\hbar }{\left|\left\langle T_{m}\right|{Ĥ}_{\mathrm{SO}}\left|S_{n}\right\rangle \right|}^{2}\delta \left(E_{S_{n}}-E_{T_{m}}\right)$$

The above equation further emphasizes the role of spin–orbit coupling (⟨T_m_|*Ĥ*_SO_|S_*n*_⟩, hereafter referred to as ζ). Analysis of the NTOs reveals a change in angular momentum associated with the S_1_ → T_3_ transition in **A–E**, which facilitates spin-state conversion.^98^ This is reflected in a slightly elevated ζ value compared to other transitions, suggesting the effect is present but not strongly pronounced. A similar trend is observed for **A–E** ^**–**^, where transitions such as S_1_ → T_1_, S_2_ → T_2_, and S_2_ → T_3_ exhibit comparable levels of coupling. Notably, markedly enhanced spin–orbit coupling is found for the S_2_ → T_1_ (ζ = 29.54 cm^–1^) and S_1_ → T_2_ (ζ = 33.35 cm^–1^) pathways. At first glance, these transitions appear to violate El-Sayed’s rule, as the involved orbitals exhibit considerable similarity in character. However, a more detailed examination of the spin–orbit coupling operator components shows that the elevated ζ values primarily originate from the *y*-component (−32.77 and −29.42 cm^–1^, respectively), which corresponds to an *n* → π* transition.

The excited-state dynamics reveal efficient ISC for **A–E**, with all transitions being highly (>90%) driven by vibronic contributions to otherwise formally forbidden transitions. The highest individual ISC rate is 1.32 × 10^10^ s^–1^ for the S_1_ → T_2_ transition. Additionaly, the T_3_ and T_1_ states are populated at rates of 2.15 × 10^9^ and 3.59 × 10^8^ s^–1^, respectively. When the slower S_1_ → T_4_ pathway is also taken into account, the total ISC rate (*k*′_ISC_, eq 8) is calculated to be 1.57 × 10^10^ s^–1^. This rate is approximately 100 times greater than the fluorescence rate (*k*_F_ = 1.53 × 10^8^ s^–1^) and comparable to the internal conversion rate (*k*_IC_ = 1.47 × 10^10^ s^–1^). As a result, the triplet state quantum yield, (Φ_T_, eq 9) for **A–E** is projected to be around 0.51, indicating a high probability of population of the triplet state following photoexcitation.

8 $$k_{\mathrm{ISC}}^{\prime }=\sum_{m=1}^{M}\sum_{n=1}^{M}k_{\mathrm{ISC}}\left(S_{n},T_{m}\right)$$

9 $$\Phi_{T}=\frac{k_{\mathrm{ISC}}^{\prime }}{k_{\mathrm{ISC}}^{\prime }+k_{F}+k_{\mathrm{IC}}}$$

For the anionic species, the analysis becomes more complex due to the involvement of the S_2_ state and inherent software limitations, such as the inability to compute the rate of S_2_ → S_1_ internal conversion pathway. Therefore, two plausible mechanistic scenarios emerge:

(1) Internal conversion dominates over intersystem crossing, leading to rapid relaxation to the S_1_ state, which then serves as the exclusive channel for triplet-state population.
(2) The S_2_ state directly contributes to triplet-state formation *via* efficient ISC. This scenario is supported by rapid singlet-to-triplet transitions, with the lowest individual *k*_ISC_ being 2.16 × 10^7^ s^–1^ for the S_2_ → T_3_ transition.

In both cases, the computed Φ_T_ are comparable: approximately 0.88 for scenario (1) and 0.89 for scenario (2). These high yields are primarily due to the exceptionally rapid ISC rates associated with the S_1_ → T_2_ (1.18 × 10^11^ s^–1^) and S_2_ → T_1_ (1.90 × 10^10^ s^–1^) transitions, consistent with their pronounced ζ values. However, considering the molar fractions of each species under physiological conditions, the apparent Φ_T_ is determined to be 0.52 in both cases, due to the negligibly low population of **A–E** ^–^.

Assuming phosphorescence is the dominant decay pathway—a reasonable assumption given the large S_0_–T_1_ energy gaps (1.80 eV for **A–E** and 1.32 eV for **A–E** ^–^) and the expected efficient energy transfer to ^3^O_2_—the singlet oxygen quantum yield (Φ_Δ_) is expected to approximate Φ_T_. Experimentally reported Φ_Δ_ values for aloe-emodin are 0.54 in acetonitrile,^40^ 0.49 in ethanol,^40^ and 0.57 in methanol,^37^ all of which surpass those of clinically approved photosensitizers such as Photofrin (Φ_Δ_ = 0.25)^99^ and Foscan (Φ_Δ_ = 0.25).^100^ This highlights aloe-emodin’s strong potential for photodynamic therapy. The assumption Φ_Δ_ ≈ Φ_T_ aligns well with the experimental data, though the actual values may be slightly lower due to nonradiative decay pathways or solvent-specific effects not fully captured in the computational model.

Finally, the overall phototoxic efficiency of a photosensitizer in Type II mechanism depends not only on triplet formation rates but also on the lifetime of the T_1_ state (τ_T_1__). Based solely on phosphorescence rates, τ_T_1__ is predicted to be 26.0 s for **A–E** and 0.66 s for **A–E** ^–^. While these lifetimes may be shortened by competing decay mechanisms, they suggest a functional balance: the neutral species allows extended interaction with oxy, whereas the anionic form exhibits more efficient ISC. This trade-off can be exploited *via* pH modulation, enabling fine-tuning of aloe-emodin's photosensitizing activity for specific therapeutic applications.

### 3.5 FEDAM Map and Type I and III Phototoxicity

While triplet–triplet annihilation is often considered as a prominent mechanism of phototoxicity, it is not the sole contributor to the overall photoreactivity of photosensitizers. The T_1_ state of a dye can also function as an autoionizer, initiating electron-transfer reactions that underpin Type I photoactivity. These reactions (eq 10–13) include

10a $$\mathrm{Ps}\left(T_{1}\right)+\mathrm{Ps}\left(S_{0}\right)\to {\mathrm{Ps}}^{•-}+{\mathrm{Ps}}^{•+}$$

10b $$\mathrm{Ps}\left(T_{1}\right)+\mathrm{Ps}\left(T_{1}\right)\to {\mathrm{Ps}}^{•-}+{\mathrm{Ps}}^{•+}$$

10c Ps(T1)+O23→Ps•++O2•−

10d Ps•−+O23→Ps(S0)+O2•−

In contrast, Type III phototoxicity involves direct oxidation of biological substrates without the involvement of oxygen. This pathway has gained interest due to its potential relevance in hypoxic environments where oxygen-mediated Type II reactivity is limited.

To assess the feasibility of the Type III pathways (eq 14), a set of biologically relevant, oxidation-prone targets was examined.^101–103^ These include five amino acids in their N-formyl forms—cysteine (Cys), histidine (His^+^), methionine (Met), tryptophan (Trp), and tyrosine (Tyr)—2′-deoxyguanine (2dG, representing nucleobase with the lowest ionization potential), and a simplified model of linoleic acid (Lin) to represent unsaturated fatty acids.

11 $$\mathrm{Ps}\left(T_{1}\right)+D\to {\mathrm{Ps}}^{•-}+D^{•+}$$

A modified version of the Full Electron Donor–Acceptor Map (FEDAM)^104^ was employed to preliminarily evaluate the viability of these pathways. The FEDAM plots vertical electron affinities (VEA) on the *X*-axis and vertical ionization potentials (VIP) on the *Y*-axis, thereby aiding in the prediction of the energetic favorability of electron transfer between the photosensitizer (in both ground and triplet states) and various targets. The resulting picture (Figure 7) provides a clear visualization of the potential photoreactive interactions relevant to Type I and Type III phototoxic mechanisms.

**Figure 7:**
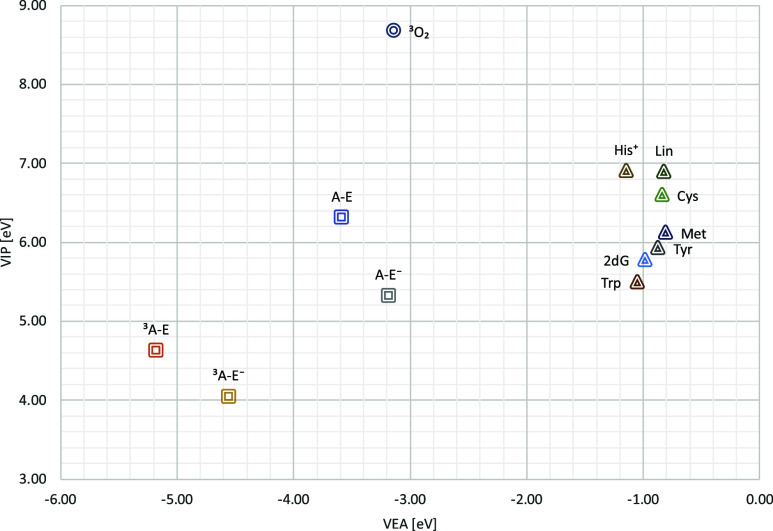
Full Electron Donor–Acceptor Map (FEDAM) for aloe–emodin species in their ground and triplet states, and potential biological targets.

Analysis of the FEDAM reveals several important features:

(a) The diagonal distance between the interacting states is slightly smaller for **A–E** ^–^ than for **A–E**, making autoionization (eq 10) more favorable for the anionic species;
(b) By summing the *X* and *Y* coordinates for the same species, both **A–E** and **A–E** ^–^ display similarly favorable behavior for triplet–triplet interactions (eq 11), with an energy value of approximately −0.55 eV;
(c) **A–E** ^–^ is more likely to generate superoxide radicals (eq 12) due to its closer proximity to the *X*-axis, indicating a lower VIP;
(d) Using the relation VEA­(S_0_) = −VIP­(Ps^•–^), it can be inferred that species positioned further below oxygen are more prone to generate superoxide radicals (eq 13). Here, **A–E** ^–^ shows a greater tendency for this pathway.
(e) The relative reactivity toward biological substrates (eq 14) is assessed by the position of the triplet state on the FEDAM. Species closer to the left side of the plot are more reactive. Based on this, **A–E** is expected to be more active in Type III photoactivity.

Table 1 summarizes the confirmation of these findings. For example, autoionization between S_0_ and T_1_ occurs more readily for **A–E** ^–^, with a rate constant of 1.51 × 10^3^ M^–1^ s^–1^, compared to 1.38 × 10^3^ M^–1^ s^–1^ for **A–E**. A dramatic difference is observed in the generation of O_2_^•–^ by T_1_: the anionic form drives this reaction with a kinetic constant of 5.04 × 10^6^ M^–1^ s^–1^—approximately 8 orders of magnitude greater than the rate for the neutral species. Furthermore, triplet–triplet annihilation occurs at higher rates for both species: 9.17 × 10^7^ M^–1^ s^–1^ for **A–E** ^–^ and even higher, at 2.53 × 10^10^ M^–1^ s^–1^, for **A–E**—approaching or exceeding the typical diffusion limit in water. For reactions between Ps^•–^ and ^3^O_2_, the computed rates are also substantial (*k*_A–E_^_–_^ = 3.64 × 10^12^ M^–1^ s^–1^; *k*_A–E_ = 7.71 × 10^10^ M^–1^ s^–1^). These results highlight that oxygen-involving pathways dominate the overall photoreactivity, which is consistent with experimental observations showing a significant reduction in phototoxicity in the absence of oxygen.^37,39,40^

**Table 1:** Vertical (Δ*E*), Adiabatic (Δ*G*), Reorganization (λ), and Activation (Δ*G* ^≠^) Energies (in kcal mol^–1^) and Reaction Rates (*k*, in M^–1^ s^–1^) for Type III Mechanisms

	Δ*E*	Δ*G*	λ	Δ*G* ^≠^	*k*
^**3**^ **A–E**					
(10a)	26.1	13.2	13.0	13.2	1.38 × 10^3^
(10b)	–12.7	–25.0	12.3	3.3	2.53 × 10^10^
(10c)	34.3	19.9	14.4	20.4	7.00 × 10^–3^
(10d)	14.5	–5.7	20.1	2.6	7.71 × 10^10^
(11)					
2dG	13.7	0.3	13.5	3.5	1.67 × 10^10^
Cys	32.8	8.3	24.5	11.0	5.66 × 10^4^
His^+^	39.7	17.7	22.0	17.9	4.46 × 10^–1^
Met	21.8	8.3	13.5	8.8	2.11 × 10^6^
Trp	7.3	–5.3	12.6	1.1	1.04 × 10^12^
Tyr	17.3	3.9	13.4	5.6	4.89 × 10^8^
Lin	39.6	17.4	22.2	17.7	6.89 × 10^–1^
^**3**^ **A–E** ^**–**^					
(10a)	17.7	11.7	6.0	13.1	1.51 × 10^3^
(10b)	–11.9	–17.2	5.3	6.6	9.17 × 10^7^
(10c)	20.7	7.8	12.9	8.3	5.04 × 10^6^
(10d)	5.0	–14.6	19.6	0.3	3.64 × 10^12^
(11)					
2dG	28.1	20.1	7.9	24.8	4.16 × 10^–6^
Cys	47.1	28.1	19.0	29.2	2.36 × 10^–9^
His^+^	54.0	37.6	16.5	44.3	2.06 × 10^–20^
Met	36.2	28.2	8.0	40.9	6.28 × 10^–18^
Trp	21.6	14.6	7.1	16.6	4.27 × 10^0^
Tyr	31.6	23.8	7.8	31.9	2.76 × 10^–11^
Lin	54.0	37.3	16.7	43.6	7.10 × 10^–20^

The feasibility of Type III pathways is limited for ^**3**^ **A–E** ^–^, which exhibits only marginal reactivity with tryptophan (*k* = 4.27 × 10^0^ M^–1^ s^–1^). In contrast, ^**3**^ **A–E** displays markedly higher reactivity: it effectively damages tryptophan and tyrosine, and—though to a lesser extent—also interacts with methionine and cysteine. Its ability to slowly oxidize cysteine (*k* = 5.66 × 10^4^ M^–1^ s^–1^) suggest potential for glutathione depletion,^41^ a mechanism that may contribute to ferroptosis induction.^105^ Importantly, neither species appears capable of efficiently oxidizing histidine, likely due to its protonated form at physiological pH. Additionally, the previously reported photoinduced lipid peroxidation^41^ is confirmed for ^**3**^ **A–E**, though the process proceeds at a slow rate (*k* = 6.89 × 10^–1^ M^–1^ s^–1^). A particularly notable observation is the high reactivity of ^**3**^ **A–E** with 2-deoxyguanosine (*k* = 1.67 × 10^10^ M^–1^ s^–1^), highlighting its capacity to induce nucleic acid lesions. This finding aligns with post-UVA-induced elevations in 8-oxoguanine levels observed in both RNA and DNA samples.^39^

### 3.6 Structure and Geometry

Although electronic differences are evident, photoexcitation and subsequent intersystem crossing do not induce significant changes in molecular geometry. The S_0_ state, the first bright excited states (S_1_ for **A–E** and S_2_ for **A–E** ^–^), and the T_1_ state are illustrated in Figure 8, along with relevant structural measurements.

**Figure 8:**
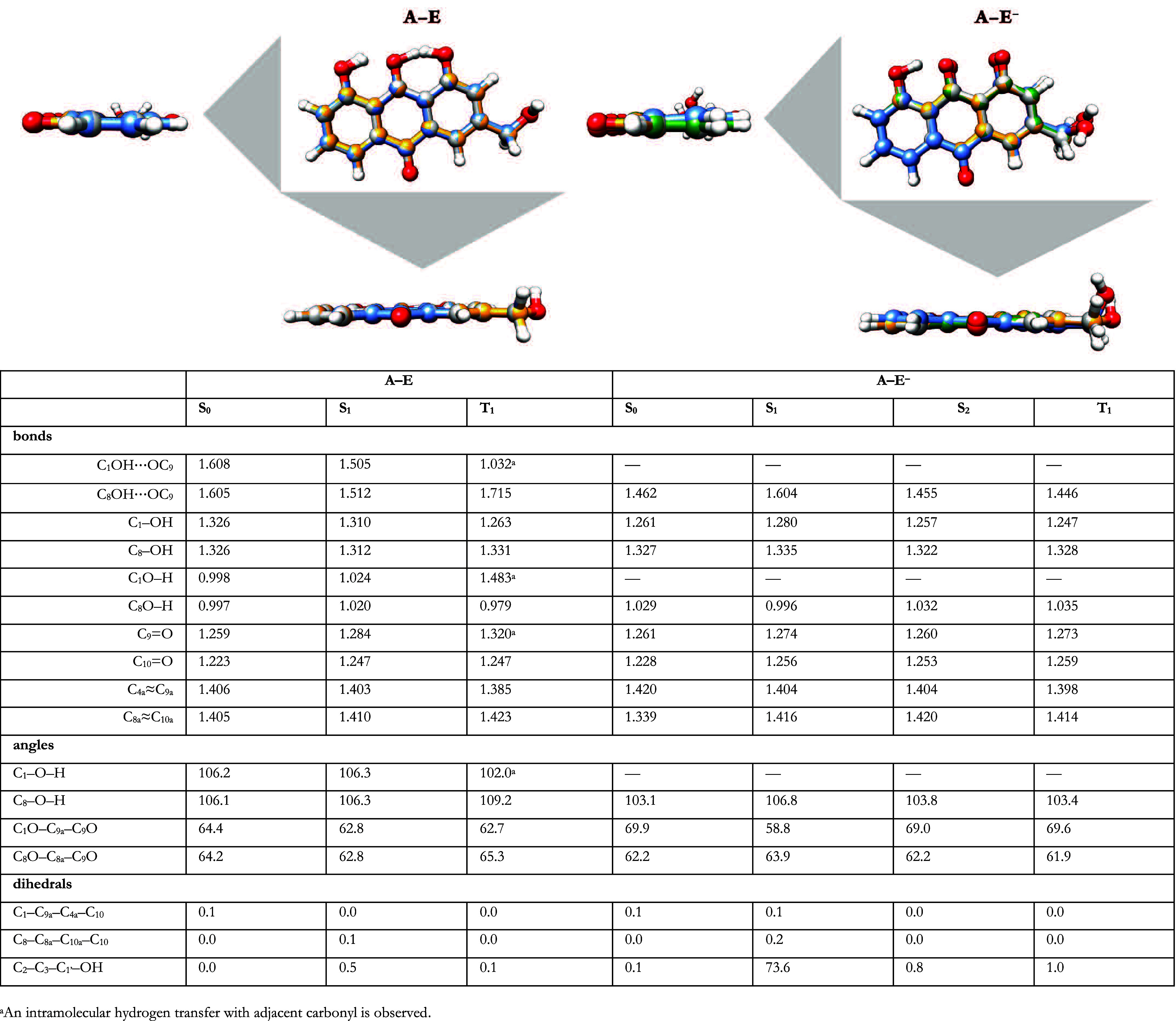
Superimposed geometries of the ground state (S_0_, gray), first excited state (S_1_, blue), second excited state (S_2_, green) and lowest-lying triplet state (T_1_, orange).

A comparison between the ground and excited states of both species reveals only minor variations in geometric parameters, with a few localized deviations. These indicate that the molecule maintains a largely rigid structure. One notable trend is the strengthening of bonds in the C ring, coupled with a simultaneous weakening of those in the A ring, as seen in alternating bond lengths. The π-delocalized electron cloud remains largely unaffected during dissociation. However, stronger repulsions are observed between the carbonyl group and the dissociated C_1_ hydroxyl in **A–E** ^–^, as reflected by the increased bond angle between them. Interestingly, this effect is not observed in the S_1_ state, where the angle decreases to 58.8°, likely due to orbital interactions, as seen in the electron natural transition orbital of **A–E** ^–^’s S_1_ state discussed previously.

For **A–E**, ISC leading to the T_1_ state induces an internal hydrogen transfer between the hydroxyl group at C1 and the adjacent carbonyl group, suggesting potential structural rearrangements upon triplet-state formation.

These structural features imply that the compound may possess the ability to intercalate with DNA, a property confirmed by experimental studies.^40^ This intercalation capability appears to be retained even upon photoexcitation. A potential structural obstacle, however, is the methoxyhydroxy residue, which, in the S_1_ state of **A–E** ^–^, is oriented 73.6° out of plane, potentially interfering with adjacent nucleotides. Given the previously assessed reactivity with 2-deoxyguanosine and the observed geometric features, further investigation into aloe–emodin’s interaction with DNA strands is warranted and will be discussed in the next section.

### 3.7 Intercalation and Photosensitization of DNA

Binding free energies for the three intercalation sites were computed for both **A–E** and **A–E** ^–^ using post-MD trajectories (Table S5). As expected, the anionic nature of DNA strands leads to significant repulsion, which is reflected in the positive electrostatic energy of all **A–E** ^–^:DNA complexes. Consequently, only **A–E** was found to effectively intercalate between the base pairs, with average total binding energies (Δ*G*_b_) of −6.6 kcal·mol^–1^ for the CG–GC site, −8.2 kcal·mol^–1^ for the AT–TA site, and −11.6 kcal·mol^–1^ for the TC–AG site. These binding energies were converted into binding constants (*K*_b_) using eq 15, yielding values of 7.1 × 10^4^, 1.0 × 10^6^, and 2.1 × 10^8^ M^–1^, respectively. Despite minor differences in simulation conditions compared to experiment—such as the inclusion of Mg^2+^ ions and a higher NaCl concentration—these computed values align well with the experimentally measured bulk DNA *K*_b_ of 8.5 × 10^5^ M^–1^.^40^

12 $$K_{b}=e^{-\Delta G_{b}/\mathrm{RT}}$$

Representative geometries further confirm that **A–E** intercalates seamlessly between base pairs, as indicated by stable RMSD plots of both the ligand and intercalated nucleotides, which fluctuate within an average range of 3–4 Å (Figure S4). Figure 9 illustrates the representative, optimized geometries of the “minimal” models (described in Section 2), alongside their corresponding RDG maps.

**Figure 9:**
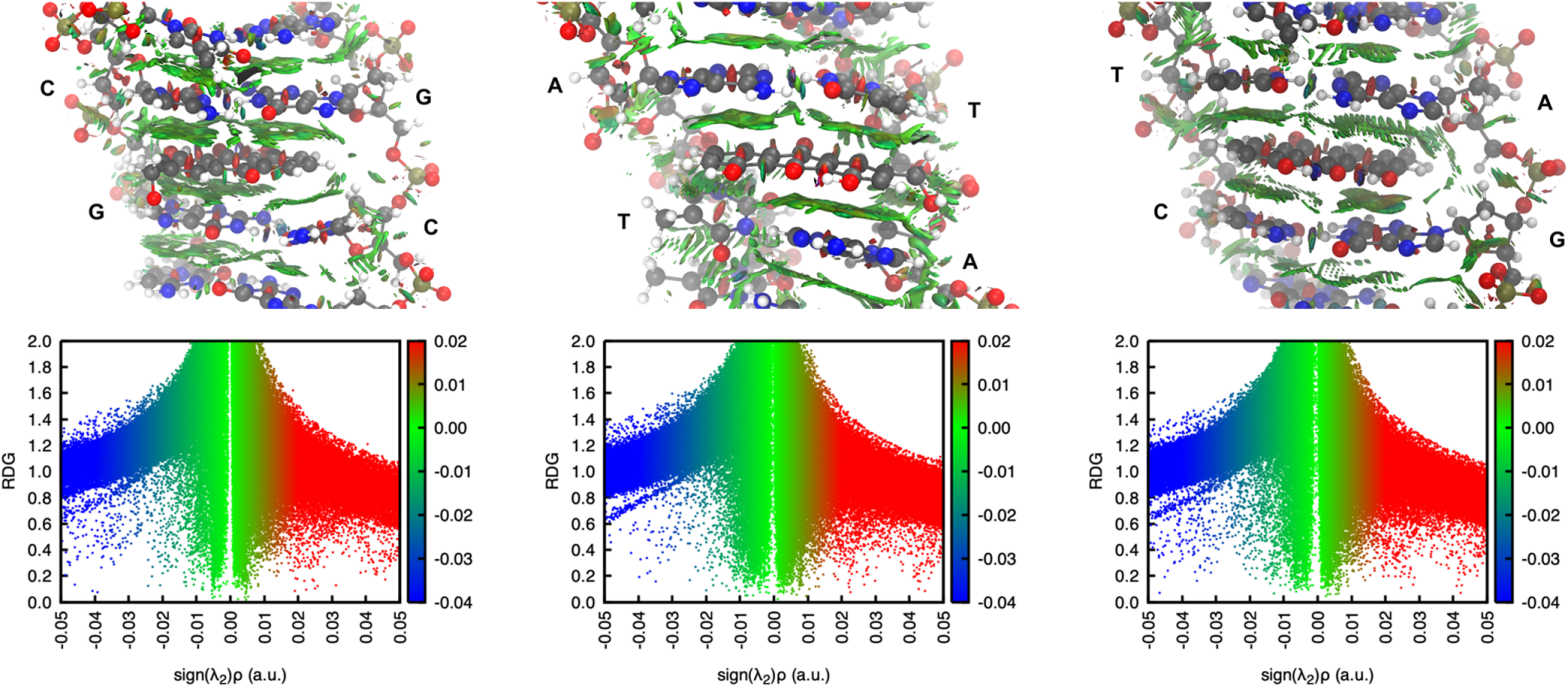
Noncovalent interaction isosurfaces (isovalue = 0.5) and reduced density gradient maps for the optimized “minimal” models of the intercalated systems.

A primary steric clash, contributing to minor instability, originates from the hydromethoxy group, which extends beyond the binding site and is oriented perpendicularly to the *Z*-axis of the DNA strand. This disruption leads to a slight elongation of the involved groove. The green isosurfaces at the intersections between the bases and the ligand correspond to favorable weak interactions, particularly π–π stacking interactions. However, neither the hydroxyl groups nor the hydromethoxy group actively participate in the intercalation process or contribute significantly to stabilization *via* hydrogen bonding.

Figure 10 presents the absorption profiles of the studied “minimal” DNA-intercalator models, with additional spectra provided in Figure S5. These results offer insights into how DNA's sensitivity to photoexcitation is altered upon intercalation.

**Figure 10:**
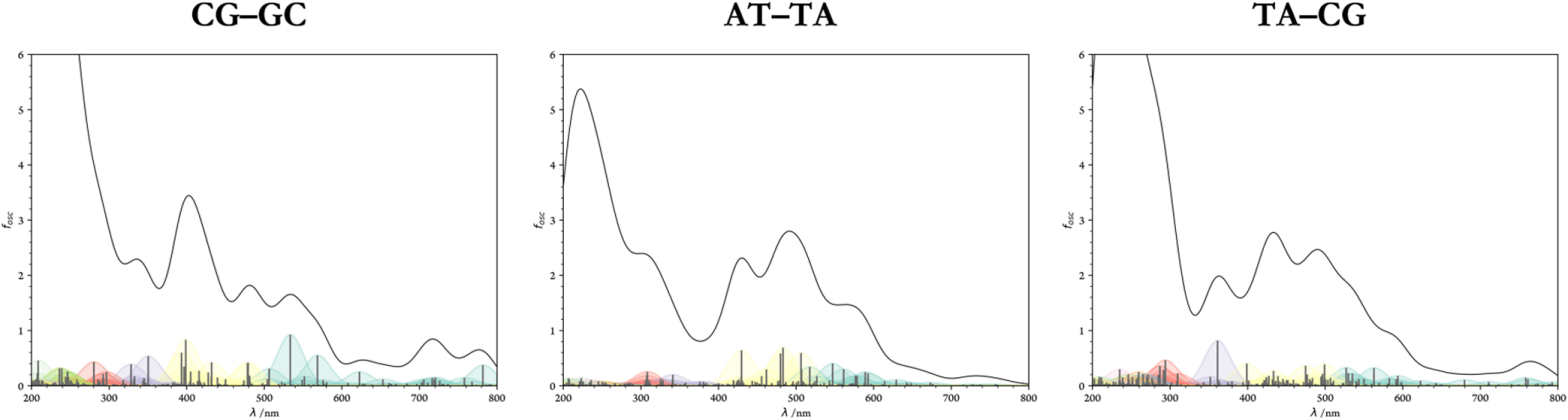
One-photon absorption spectra of the “minimal” DNA-intercalator models.

At the outset, the spectra of unperturbed DNA fragments suggest that intercalation induces notable changes, altering absorption profiles by increasing intensities and ultimately making DNA more susceptible to direct photodamage. However, even without functioning as a direct photosensitizer, **A–E** may still exhibit cytotoxic effects by inducing significant geometric distortions in nucleotides and destabilizing DNA, as evidenced by the preceding figures and spectra. These structural rearrangements could potentially lead to delayed cell cycle arrest and, ultimately, apoptosis over extended periods.

Notably, new peaks near the absorption region of the free ligand can be identified by referencing the known absorption spectrum of **A–E**. By comparing the spectra presented above with those of the non-relaxed systems—i.e., after ligand removal, isolating spectral changes solely due to **A–E**'s presence—the newly emergent peak at approximately 475 nm (highlighted in yellow) in the AT–TA system can be clearly attributed to the ligand. This effect is less pronounced in the TA–CG system, where no distinct single peak appears; instead, a general increase in absorption intensity is observed within the 475–575 nm range, along with a smoother shoulder. In the CG–GC system, the effect is even less pronounced.

Given that the experimentally determined binding constant—measured using fluorescence spectroscopy^40^—closely aligns with the computed *K*_b_ for the AT–TA site, it is likely that this specific site predominantly contributes to the observed experimental behavior. The changes in absorption spectra at other intercalation sites are less pronounced, consistent with previous reports of fluorescence quenching.^39,40^

## 4 Conclusions

This study employed a comprehensive set of computational approaches to investigate the photophysics of aloe-emodin, correlating closely with available experimental data. At physiological pH, **A–E** and **A–E^–^** exist in a 98:2 ratio. The computed absorption spectra closely match experimental findings and reveal that deprotonation leads to a bathochromic shift due to changes in HOMO ordering, while also rendering the S_1_ state dark. Nonetheless, the S_2_ excitation of **A–E** ^–^ requires less energy than the S_1_ excitation of **A–E**.

Aloe-emodin exhibits strong potential as a two-photon photosensitizer for photodynamic therapy, with its highly conjugated π-electron system enabling two-photon absorption at the relevant scale. The most efficient transition for **A–E** is the S_0_ → S_3_ excitation at 802.5 nm, displaying high TPA cross section value of 93.7 GM for linear polarization. For **A–E** ^–^, TPA transitions are even stronger. The results suggest meaningful photoexcitation making aloe-emodin suitable for TP-PDT, thereby drawing advantages from tissue penetration and uniform activation.

The photophysical properties of aloe-emodin were thoroughly analyzed, with an almost complete reconstruction of its excited-state dynamics, resulting in the development of Jablonski diagrams for both species. The quantum yield of triplet formation was approximately 0.52 for **A–E**, while **A–E** ^–^ exhibiting significantly higher ISC rates, yielded near-complete ISC efficiency and an average quantum yield of 0.89 (0.88 excluding S_2_ participation). Although the lowest-lying triplet states possess modest lifetimes, they actively participate in autoionization and generate superoxide anion radicals. While ^**3**^ **A–E** ^**–**^ shows selective reactivity, oxidizing only tryptophan, ^**3**^ **A–E** exhibits broader reactivity, engaging with all tested amino acids except histidine. Additionaly, ^**3**^ **A–E** is capable of inducing DNA damage and may also initiate lipid peroxidation at a slow rate.

Due to the geometric rigidity of aloe-emodin in both ground and excited states, its potential for DNA intercalation was further explored. As expected, repulsive interactions between the negatively charged **A–E** ^–^ and DNA hinder binding. In contrast, **A–E** was found to intercalate favorably at the examined sites. UV–VIS spectral analysis indicates that intercalation at the AT–TA site induces a distinct absorption peak, whereas other sites show less pronounced spectral changes. However, local nucleotide rearrangements at these sites led to variations in the absorption curves.

In conclusion, the results of this study complement existing experimental findings^37,39–41^ by providing atomistic insights that enhance understanding of aloe-emodin’s photoproperties. The computational protocol demonstrated here has proven to be reliable and is expected to be applicable to other 9,10-anthraquinones, supporting further research into their biological activity and photophysical properties.

## Supplementary Material



## Notes

Details on acid–base equilibria; OPA and TPA benchmarks; absorption peak energies (in nm) for the first bright states; average energy differences (complex–receptor–ligand, in kcal mol^–1^) estimated with MMPBSA over total production time; theoretical UV–VIS absorption spectra for the tested functionals; evolution of the root–mean square deviation (RMSD) over 100 ns of simulation, and *xyz*-coordinates with energies (PDF)
