# Supplementary material for: On the Photosensitizing Properties of Aloe-Emodin in Photodynamic Therapy: Insights from the Molecular Modeling
Source: J Phys Chem B. 2025 May 31;129(23):5683–97. doi: 10.1021/acs.jpcb.5c01117 (PMC12169682; doi:10.1021/acs.jpcb.5c01117)
Supplement: Supplementary file 1 [file jp5c01117_si_001.pdf]

On the Photosensitizing Properties of Aloe-Emodin in Photodynamic Therapy: Insights from the Molecular Modeling

Maciej Spiegel

Department of Organic Chemistry and Pharmaceutical Technology, Faculty of Pharmacy, Wroclaw Medical University, Borowska 211A, 50-556 Wroclaw, Poland

1. ACID–BASE EQUILIBRIA

The initial conformation of neutral aloe-emodin was generated using '*an efficient scheme for in silico sampling of parts of the molecular chemical space by semiempirical tight-binding methods combined with a meta-dynamics-driven search algorithm*'[1], as implement in the *CREST* program[2]. Implicit solvation was modeled using the analytical linearized Poisson–Boltzmann model[3], and a preliminary geometry optimization was carried out using the GFN2–xTB method.[4]

Given the polyphenolic nature of aloe-emodin, its dissociation constants were estimated using the fitted parameters method[5], and the resulting values were used to compute molar fractions of each species at physiological pH. To achieve this, the lowest-energy geometry produced by *CREST* was reoptimized in *Gaussian 16 (rev. C.02)*[6], at the PBE0[7,8]/6–311+G(d,p)[9,10] level of theory. This approach is recognized as "*benchmark–tier*" by Grimme *et al.*[11] and has been shown to produce low errors in the pK_a estimation methodology employed here.[5] Solvation in water was modeled using the Universal Solvation Model Based on Solute Electron Density (SMD).[12]

From the optimized neutral structure, two possible monoanions—resulting from deprotonation at either the hydroxyl group on C1 or C8—were generated and optimized. This procedure was repeated for the most stable monoanion to generate the corresponding dianion. Following the above referenced method, pK_a values and molar fractions (**Figure S2**) were assessed.

2. ONE–PHOTON ABSORPTION BENCHMARK

The *CREST*-generated conformation was reoptimized in *Orca 6.0*[13] under the same stringent using the PBE0[7,8] functional (see reasoning above), paired with the ma–def2–TZVP basis set, as discussed in the main text. The SMD solvation model was used to simulate acetonitrile, in line with the experimental conditions under which the spectra were recorded.[14] Notably, the first bright states reported in acetonitrile, methanol[15,16], ethanol[17], and phosphate-buffered saline[18] were all found to be energetically almost identical.

Absorption spectra for the optimized structures were computed using 19 different functionals spanning various levels of Perdew's Jacob's Ladder and including: B3LYP (*Turbomole* version)[19–21], B3LYP/G (*Gaussian* version), B3PW91, B97[22,23], BHANDHLYP, CAM–B3LYP[24], LC–BLYP[25], LC–PBE[26], M06L[27,28], O3LYP[29,30], PBE0[7,8], revTPSS, TPSS[31,32], TPSSh[33], ωB97[34], ωB97X[34], X3LYP[35]. The results were compared with experimental data (**Tables S1**), with the corresponding spectra shown in **Figure S4**.

The data indicate that the best-performing functional is O3LYP, showing minimal deviation (ΔE = 5.3 nm) from the experimentally measured absorption maximum at ~430 nm.

For the M06L[27], M06[28], and M06-2X[28] functionals, spectra could not be generated in a regular manner due to linear dependency issues with the chosen basis set. However, increasing the integration grid enabled Davidson diagonalization to proceed. The resulting first bright-state energies were 443.0 nm, 397.0 nm, and 347.5 nm, respectively. While M06L provided the most reasonable outcome among the Minnesota functionals, its performance still fell short compared to O3LYP. For consistency, the O3LYP spectrum was regenerated using the same enhanced integration grid, yielding a first bright-state transition at 434.9 nm—bringing it even closer to the experimental reference. This further confirmed the superior performance of Handy's functional in this context.

3. TWO–PHOTON ABSORPTION BENCHMARK

A benchmark study of two–photon absorption properties was conducted for emodin, for which an experimental TPA cross-section (σ_{TPA}) of 380.9 GM was reported in methanol under an 800 nm two-photon laser (λ_{TPA})[36]. Following the methodology described in the previous section, the lowest-energy conformation was generated using *CREST* and subsequently reoptimized at the PBE0/ma-def2-TZVP level of theory within the SMD solvation model for methanol. The resulting optimized structures were subsequently employed in TPA calculations using the *Dalton* program, applying functionals that are fully implemented or validated for quadratic response theory—SVWN5, BVWN, BHandH, BHandHLYP, CAM–B3LYP, B1LYP, and B3LYP—in conjunction with the aug-cc-pVDZ basis set.

For all tested functionals, the $S_0 \rightarrow S_3$ transition corresponds to the highest σ_{TPA}. The simplest functional, SVWN5, significantly overestimates the property. Its strongest σ_{TPA} of 63.2 GM appears at 939.3 nm and fails to reproduce absorption

near the experimental wavelength, instead predicting transitions at 837.7 nm and 756.0 nm with negligible or low cross-sections.

Introducing Becke's exchange, as in BVWN, does not markedly improve performance—the highest σ_{TPA} is 64.9 GM, at a wavelength approximately 130 nm higher than the reference value. Swapping the VWN correlation part for LYP, as in BLYP, also yields no significant changes, with results remaining largely consistent with those from BVWN.

In contrast, the half-and-half hybrid functionals BHandH and BHandHLYP underestimate σ_{TPA} and predict maximum activity at shorter wavelengths—672.0 nm and 668.4 nm—with cross-sections of 66.6 GM and 64.6 GM, respectively.

A particularly relevant observation is that CAM-B3LYP, though often recommended for TPA studies, performs poorly for this system. Its highest σ_{TPA} of 68.9 GM is significantly blue-shifted relative to the experimental λ_{TPA}, appearing nearly 120 nm below it at 688.8 nm. This highlights the necessity of system-specific benchmarking.

The most satisfactory results were obtained using B1LYP and B3LYP. For both, the strongest TPA transitions correspond to σ_{TPA} values of 72.4 GM (B1LYP) and 72.3 GM (B3LYP). Their cross-sections are only marginally different from CAM-B3LYP, while they offer a significantly better match with the experimental wavelength—while B1LYP slightly underestimates λ_{TPA} (767.7 nm), B3LYP offers excellent agreement (794.8 nm).

Therefore, among the tested functionals, B3LYP emerges as the most reasonable choice—particularly since *Dalton* does not currently support O3LYP. Structurally, O3LYP differs from B3LYP primarily by replacing B88 with OPTX, slightly adjusting the mixing coefficients, substituting VWN with VWN5, and reducing the Hartree–Fock exchange fraction by 8%.[37]. Although B3LYP underestimates the experimental σ_{TPA} by more than a factor of five, it is important to note that the experimental value is relative to Rhodamine B and may be affected by sample concentration, π–π stacking of delocalized systems, or other experimental conditions. Conversely, current theoretical methods are known to struggle with the accurate prediction of TPA profiles.

In conclusion, the computed λ_{TPA} of 794.8 nm and σ_{TPA} of 72.3 GM for emodin in methanol should not be considered incorrect but should be interpreted with caution, ideally for comparative purposes. Notably, when recalculated for water as the solvent, the λ_{TPA} remains the same, while the σ_{TPA} increases slightly to 75.3 GM.

4. SUPPLEMENTARY TABLES

Table S1. Absorption peak energies (in nm) for the first bright states.

Meta-GGA		*RSH GGA*	
**M06L**	—†	**CAM-B3LYP**	356.8
**TPSS**	463.9	**LC-BLYP**	344.4
**revTPSS**	461.8	**LC-PBE**	314.2
GH-GGA		**ωB97**	323.2
**B3LYP**	410.2	**ωB97X**	334.4
**B3LYP/G**	410.2	*GH meta-GGA*	
**B3PW91**	408.9	**M06**	—†
**B97**	410.3	**M06-2X**	—†
**BHANDHLYP**	341.8	**TPSSh**	435.8
**O3LYP**	435.3	*Reference*[14]	*430*
**PBE0**	395.5		
**X3LYP**	405.5		

†The regular job failed at Davidson-Diagonalization due to the linear dependency with the chosen basis set.

Table S2. Absorption wavelengths (λ, nm), excitation energies (E, eV), oscillator strengths (f), and main electronic configuration for the examined compounds in water solvent, calculated at the O3LYP/ma-def2-TZVP level of theory.

	**Exct.**	**λ**	**E**	**f**	**Composition**
**A-E**	S_1	454.9	2.73	0.354	H→L (94.73%)
	S_2	438.3	2.83	0.000	H-2 → L (98.04%)
	S_3	396.4	3.13	0.008	H-1 → L (93.38%)
	S_4	378.7	3.27	0.013	H-3 → L (93.41%)
	S_5	360.8	3.44	0.000	H-5 → L (97.58%)
**A-E⁻**	S_1	581.7	2.13	0.000	H-1 → L (99.33%)
	S_2	532.9	2.33	0.294	H → L (92.37%)
	S_3	407.4	3.04	0.036	H-2 → L (89.55%)
	S_4	393.6	3.15	0.000	H-4 → L (96.12%)
	S_5	373.1	3.32	0.037	H-3 → L (88.49%)

Table S3. Excitation energies (E, in eV), effective photon wavelengths (λ_{TPA}, in nm, calculated as half of ΔE), and two-photon cross sections (σ_{TPA}, in GM) under linearly polarized laser beam of emodin established with different functionals coupled with aug-cc-pVDZ basis set.

S→S$_N$	E	λ_{TPA}	σ_{TPA}	E	λ_{TPA}	σ_{TPA}	E	λ_{TPA}	σ_{TPA}	E	λ_{TPA}	σ_{TPA}
B1LYP				*BHandHLYP*			*CAM–B3LYP*			*SVWN5*		
1	2.90	855.1	0.1	3.39	731.5	0.3	3.23	767.7	0.3	2.33	1064.2	0.3
2	3.18	779.8	0.0	3.67	675.7	0.0	3.40	729.3	0.0	2.44	1016.3	0.0
3	3.23	767.7	72.4	3.71	668.4	64.6	3.60	688.8	68.9	2.64	939.3	63.2
4	3.42	725.1	27.3	3.94	629.4	27.0	3.85	644.1	26.9	2.74	905.0	24.6
5	3.79	654.3	0.0	4.38	566.1	0.0	4.08	607.8	0.0	2.96	837.7	0.0
6	4.01	618.4	12.0	4.56	543.8	9.2	4.47	554.7	12.8	3.28	756.0	14.4
B3LYP				*BLYP*			*BVWN*			*BHandH*		
1	2.79	888.8	0.1	2.36	1050.7	0.3	2.38	1041.9	0.3	3.36	738.0	0.3
2	3.06	810.4	0.0	2.52	984.0	0.0	2.55	972.4	0.0	3.59	690.7	0.0
3	3.12	794.8	72.3	2.66	932.2	64.9	2.67	928.7	64.9	3.69	672.0	66.6
4	3.30	751.4	26.6	2.77	895.2	22.9	2.78	892.0	22.6	3.93	631.0	27.6
5	3.65	679.4	0.0	3.01	823.8	0.0	3.03	818.4	0.0	4.32	574.0	0.0
6	3.88	639.1	12.5	3.32	746.9	11.7	3.33	744.6	11.5	4.55	545.0	10.8

Table S4. Excitation energies (E, in eV), effective photon wavelengths (λ_{TPA}, in nm, calculated as half of ΔE), transition probabilities (δ_{TPA}, in a.u.), and two-photon cross sections (σ_{TPA}, in GM) under linearly polarized laser beam of **A–E** and **A–E⁻** computed at B3LYP/aug-cc-pVDZ level of theory.

S→S$_N$	E	λ_{TPA}	δ_{TPA}	σ_{TPA}
**A–E**				
1	2.69	921.8	0.323×10^{3}	1.3
2	2.98	832.1	0.352×10^{1}	0.0
3	3.09	802.5	0.181×10^{5}	93.7
4	3.41	721.2	0.262×10^{4}	16.5
5	3.67	675.7	0.201×10^{4}	0.0
6	4.14	599.9	0.638×10^{4}	67.4
**A–E⁻**				
1	2.12	1169.7	0.771×10^{4}	17.1
2	2.29	1082.8	0.125×10^{1}	0.0
3	3.07	807.7	0.346×10^{5}	176.3
4	3.26	760.6	0.219×10^{0}	0.0
5	3.45	718.7	0.333×10^{4}	21.5
6	3.65	679.4	0.425×10^{4}	0.0
7	3.69	67Fo2.0	0.285×10^{5}	210.5

Table S5. Average energy differences (Complex – Receptor – Ligand, in kcal mol^{-1}) estimated with MMPBSA over total production time.

	A-E			A-E⁻		
	ATTA	CGGC	TCAG	ATTA	CGGC	TCAG
$E_{VDWAALS}$	–38.4359	- 39.3631	-40.1352	-38.8276	- 42.0466	- 38.1912
E_{EEL}	–1.6715	-4.4730	-7.1574	557.2622	503.4641	546.5162
E_{PB}	18.2736	23.1297	21.8170	-519.9140	- 471.0854	- 505.8330
E_{NPOLAR}	–19.9966	-20.0728	-20.9378	-19.8711	- 20.5882	- 20.2754
E_{DISPER}	33.6350	34.1749	34.7745	36.8905	34.4579	32.4361
ΔG_{gas}	–40.1074	-43.8361	-47.2926	518.4346	461.4175	508.3250
ΔG_{solv}	31.9119	37.2318	35.6538	- 502.8946	- 457.2158	- 493.6723
ΔTOTAL	-8.1954	-6.6043	-11.6388	15.5400	4.2018	14.6527

5. SUPPLEMENTARY FIGURES

TPSS

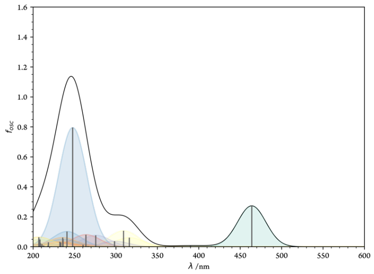

revTPSS

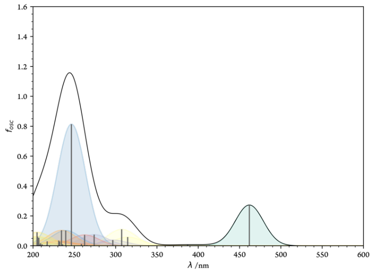

B3LYP

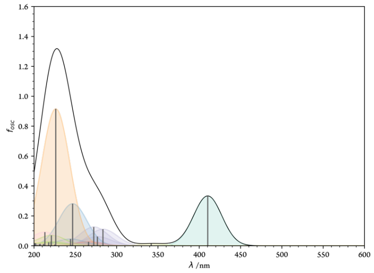

B3LYP/G

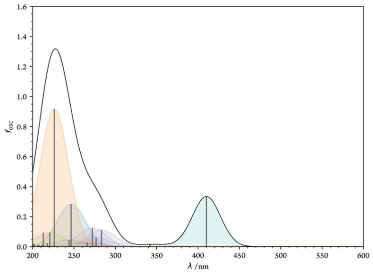

B3PW91

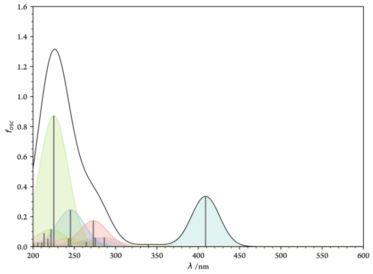

B97

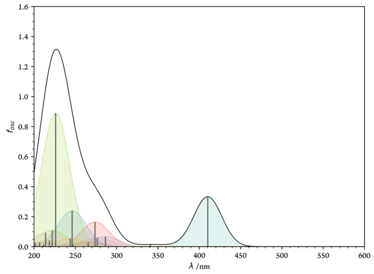

BHANDHLYP

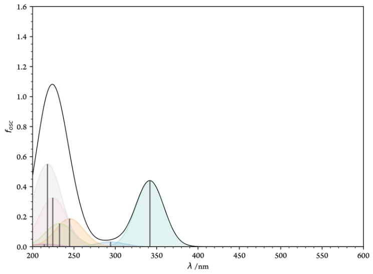

O3LYP

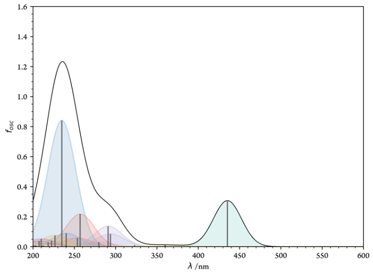

PBE0

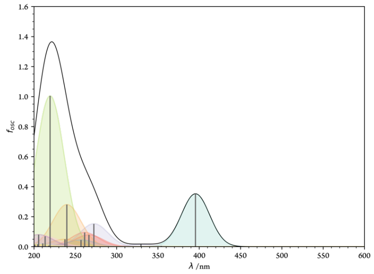

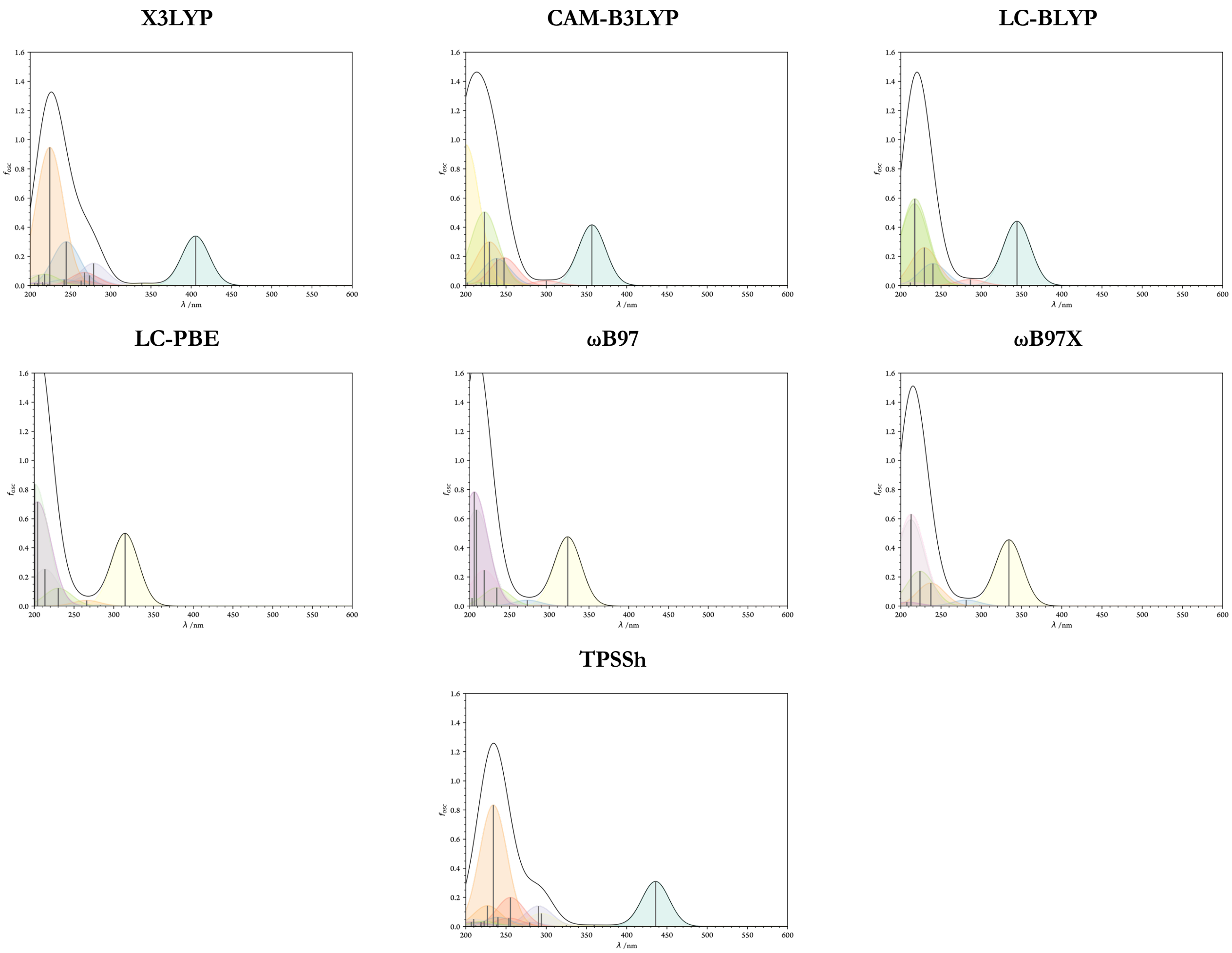

Figure S1. Theoretical UV–VIS absorption spectra for the tested functionals.

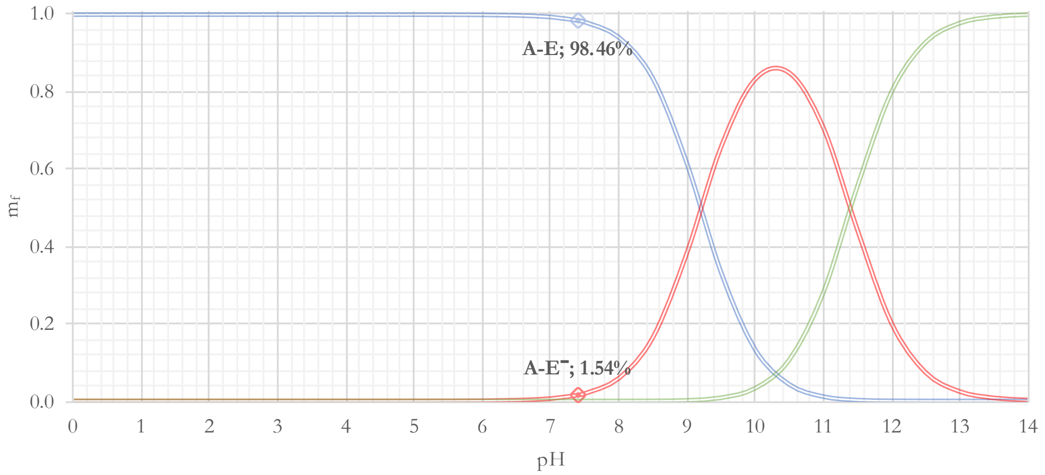

Figure S2. Molar fraction of aloe–emodin species as function of pH.

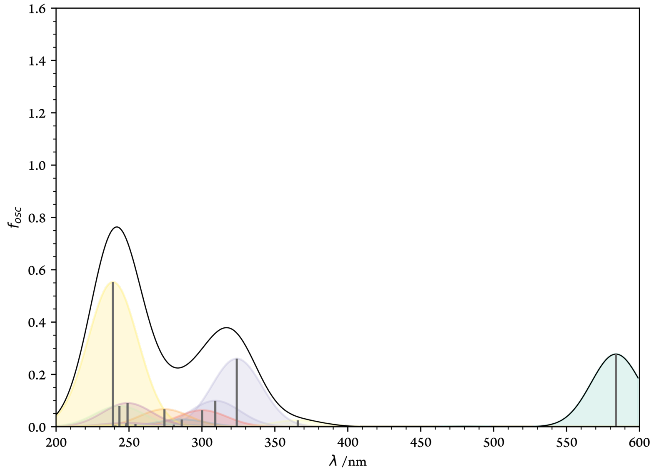

Figure S3. One–photon absorption spectra of **A–E^{2-}**.

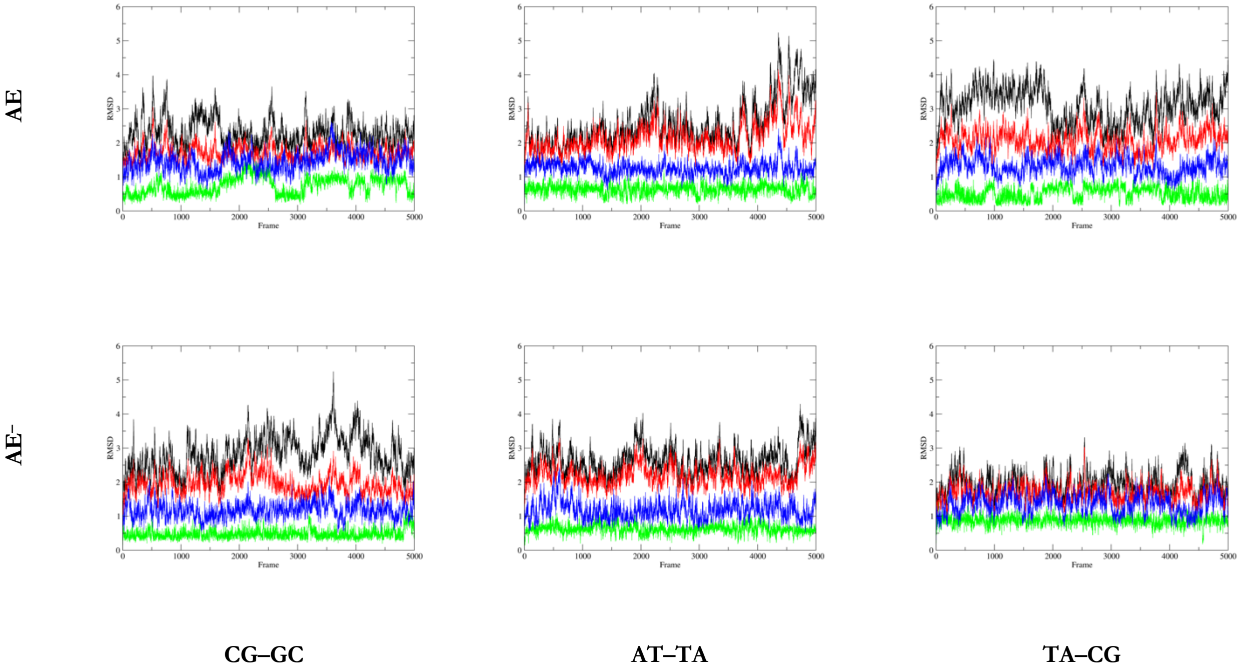

Figure S4. Evolution of the root–mean square deviation (RMSD) over 100 ns of simulation. The black line represents the entire system, the red line corresponds to the system excluding labile terminal residues on each strand, the blue line represents the nucleotides in direct vicinity to the ligand, and the green line corresponds to the ligand only.

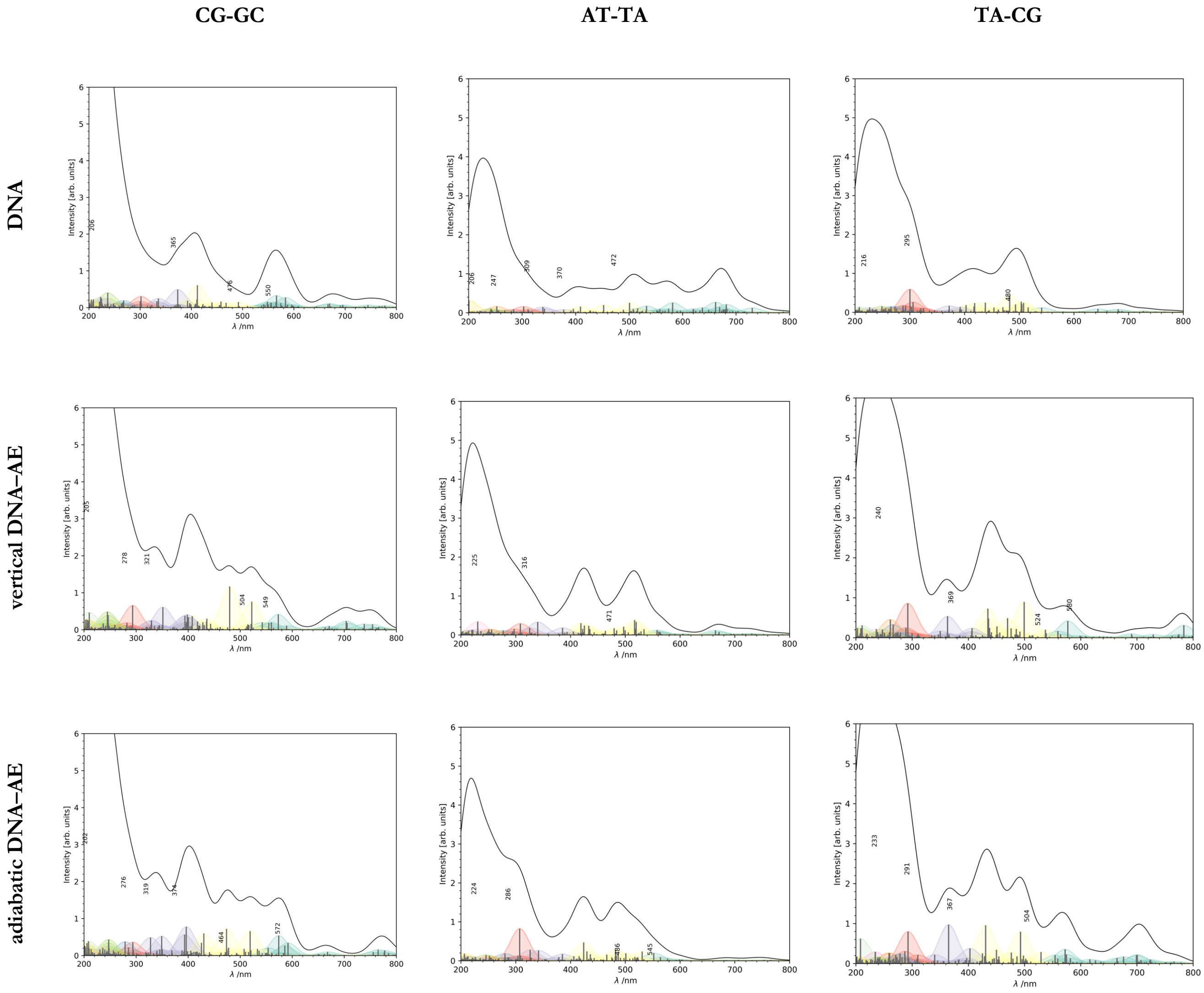

Figure S5. Absorption spectra of the '*minimal*' models at different stages of the intercalation process. '*DNA*' refers to the '*minimal*' model before intercalation; '*vertical DNA–AE*' represents the model after ligand removal without reoptimization; and '*adiabatic DNA–AE*' corresponds to the optimized model after ligand deletion.

6. REFERENCES

(1) Pracht, P.; Bohle, F.; Grimme, S. Automated Exploration of the Low-Energy Chemical Space with Fast Quantum Chemical Methods. *Phys. Chem. Chem. Phys.* **2020**, *22* (14), 7169–7192. https://doi.org/10.1039/C9CP06869D.

(2) Pracht, P.; Grimme, S.; Bannwarth, C.; Bohle, F.; Ehlert, S.; Feldmann, G.; Gorges, J.; Müller, M.; Neudecker, T.; Plett, C.; Spicher, S.; Steinbach, P.; Wesołowski, P. A.; Zeller, F. CREST—A Program for the Exploration of Low-Energy Molecular Chemical Space. *The Journal of Chemical Physics* **2024**, *160* (11), 114110. https://doi.org/10.1063/5.0197592.

(3) Ehlert, S.; Stahn, M.; Spicher, S.; Grimme, S. Robust and Efficient Implicit Solvation Model for Fast Semiempirical Methods. *J. Chem. Theory Comput.* **2021**, *17* (7), 4250–4261. https://doi.org/10.1021/acs.jctc.1c00471.

(4) Grimme, S. Exploration of Chemical Compound, Conformer, and Reaction Space with Meta-Dynamics Simulations Based on Tight-Binding Quantum Chemical Calculations. *J. Chem. Theory Comput.* **2019**, *15* (5), 2847–2862. https://doi.org/10.1021/acs.jctc.9b00143.

(5) Galano, A.; Pérez-González, A.; Castañeda-Arriaga, R.; Muñoz-Rugeles, L.; Mendoza-Sarmiento, G.; Romero-Silva, A.; Ibarra-Escutia, A.; Rebollar-Zepeda, A. M.; León-Carmona, J. R.; Hernández-Olivares, M. A.; Alvarez-Idaboy, J. R. Empirically Fitted Parameters for Calculating p *K* a Values with Small Deviations from Experiments Using a Simple Computational Strategy. *J. Chem. Inf.*

Model. **2016**, *56* (9), 1714–1724. https://doi.org/10.1021/acs.jcim.6b00310.
(6) Frisch, M. J.; Trucks, G. W.; Schlegel, H. B.; Scuseria, G. E.; Robb, M. A.; Cheeseman, J. R.; Scalmani, G.; Barone, V.; Petersson, G. A.; Nakatsuji, H.; Li, X.; Caricato, M.; Marenich, A. V.; Bloino, J.; Janesko, B. G.; Gomperts, R.; Mennucci, B.; Hratchian, H. P.; Ortiz, J. V.; Izmaylov, A. F.; Sonnenberg, J. L.; Williams-Young, D.; Ding, F.; Lipparini, F.; Egidi, F.; Goings, J.; Peng, B.; Petrone, A.; Henderson, T.; Ranasinghe, D.; Zakrzewski, V. G.; Gao, J.; Rega, N.; Zheng, G.; Liang, W.; Hada, M.; Ehara, M.; Toyota, K.; Fukuda, R.; Hasegawa, J.; Ishida, M.; Nakajima, T.; Honda, Y.; Kitao, O.; Nakai, H.; Vreven, T.; Throssell, K.; Montgomery, J. A., Jr.; Peralta, J. E.; Ogliaro, F.; Bearpark, M. J.; Heyd, J. J.; Brothers, E. N.; Kudin, K. N.; Staroverov, V. N.; Keith, T. A.; Kobayashi, R.; Normand, J.; Raghavachari, K.; Rendell, A. P.; Burant, J. C.; Iyengar, S. S.; Tomasi, J.; Cossi, M.; Millam, J. M.; Klene, M.; Adamo, C.; Cammi, R.; Ochterski, J. W.; Martin, R. L.; Morokuma, K.; Farkas, O.; Foresman, J. B.; Fox, D. J. Gaussian˜16 Revision C.01, 2016.
(7) Ernzerhof, M.; Scuseria, G. E. Assessment of the Perdew–Burke–Ernzerhof Exchange-Correlation Functional. *The Journal of Chemical Physics* **1999**, *110* (11), 5029–5036. https://doi.org/10.1063/1.478401.
(8) Adamo, C.; Barone, V. Toward Reliable Density Functional Methods without Adjustable Parameters: The PBE0 Model. *The Journal of Chemical Physics* **1999**, *110* (13), 6158–6170. https://doi.org/10.1063/1.478522.
(9) Krishnan, R.; Binkley, J. S.; Seeger, R.; Pople, J. A. Self-Consistent Molecular Orbital Methods. XX. A Basis Set for Correlated Wave Functions. *The Journal of Chemical Physics* **1980**, *72* (1), 650–654. https://doi.org/10.1063/1.438955.
(10) Clark, T.; Chandrasekhar, J.; Spitznagel, G. W.; Schleyer, P. V. R. Efficient Diffuse Function-augmented Basis Sets for Anion Calculations. III. The 3-21+G Basis Set for First-row Elements, Li–F. *J Comput Chem* **1983**, *4* (3), 294–301. https://doi.org/10.1002/jcc.540040303.
(11) Bursch, M.; Mewes, J.; Hansen, A.; Grimme, S. Best-Practice DFT Protocols for Basic Molecular Computational Chemistry**. *Angew Chem Int Ed* **2022**, *61* (42), e202205735. https://doi.org/10.1002/anie.202205735.
(12) Marenich, A. V.; Cramer, C. J.; Truhlar, D. G. Universal Solvation Model Based on Solute Electron Density and on a Continuum Model of the Solvent Defined by the Bulk Dielectric Constant and Atomic Surface Tensions. *J. Phys. Chem. B* **2009**, *113* (18), 6378–6396. https://doi.org/10.1021/jp810292n.
(13) Neese, F. Software Update: The ORCA Program System—Version 5.0. *WIREs Comput Mol Sci* **2022**, *12* (5), e1606. https://doi.org/10.1002/wcms.1606.
(14) Vath, P.; Wamer, W. G.; Falvey, D. E. Photochemistry and Phototoxicity of Aloe Emodin. *Photochemistry and Photobiology* **2002**, *75* (4), 346–352. https://doi.org/10.1562/0031-8655(2002)0750346PAPOAE2.0.CO2.
(15) Francis, W. R.; Powers, M. L.; Haddock, S. H. D. Characterization of an Anthraquinone Fluor from the Bioluminescent, Pelagic Polychaete *Tomopteris*. *Luminescence* **2014**, *29* (8), 1135–1140. https://doi.org/10.1002/bio.2671.
(16) Zang, L.; Zhao, H.; Ji, X.; Cao, W.; Zhang, Z.; Meng, P. Photophysical Properties, Singlet Oxygen Generation Efficiency and Cytotoxic Effects of Aloe Emodin as a Blue Light Photosensitizer for Photodynamic Therapy in Dermatological Treatment. *Photochem Photobiol Sci* **2017**, *16* (7), 1088–1094. https://doi.org/10.1039/c6pp00453a.
(17) Vargas, F.; Fraile, G.; Velasquez, M.; Correia, H.; Fonseca, G.; Marin, M.; Marcano, E.; Sánchez, Y. Studies on the Photostability and Phototoxicity of Aloe-Emodin, Emodin and Rhein. *Die Pharmazie* **2002**, *57* (6), 399–404.
(18) Wamer, W. G.; Vath, P.; Falvey, D. E. In Vitro Studies on the Photobiological Properties of Aloe Emodin and Aloin A. *Free Radical Biology and Medicine* **2003**, *34* (2), 233–242. https://doi.org/10.1016/S0891-5849(02)01242-X.
(19) Becke, A. D. Density-Functional Thermochemistry. III. The Role of Exact Exchange. *The Journal of Chemical Physics* **1993**, *98* (7), 5648–5652. https://doi.org/10.1063/1.464913.
(20) Becke, A. D. Density-Functional Exchange-Energy Approximation with Correct Asymptotic Behavior. *Phys. Rev. A* **1988**, *38* (6), 3098–3100. https://doi.org/10.1103/PhysRevA.38.3098.
(21) Lee, C.; Yang, W.; Parr, R. G. Development of the Colle-Salvetti Correlation-Energy Formula into a Functional of the Electron Density. *Phys. Rev. B* **1988**, *37* (2), 785–789. https://doi.org/10.1103/PhysRevB.37.785.
(22) Schmider, H. L.; Becke, A. D. Optimized Density Functionals from the Extended G2 Test Set. *The Journal of Chemical Physics* **1998**, *108* (23), 9624–9631. https://doi.org/10.1063/1.476438.
(23) Becke, A. D. Density-Functional Thermochemistry. V. Systematic Optimization of Exchange-Correlation Functionals. *The Journal of Chemical Physics* **1997**, *107* (20), 8554–8560. https://doi.org/10.1063/1.475007.
(24) Yanai, T.; Tew, D. P.; Handy, N. C. A New Hybrid Exchange–Correlation Functional Using the Coulomb-Attenuating Method (CAM-B3LYP). *Chemical Physics Letters* **2004**, *393* (1–3), 51–57. https://doi.org/10.1016/j.cplett.2004.06.011.
(25) Tawada, Y.; Tsuneda, T.; Yanagisawa, S.; Yanai, T.; Hirao, K. A Long-Range-Corrected Time-Dependent Density Functional Theory. *The Journal of Chemical Physics* **2004**, *120* (18), 8425–8433. https://doi.org/10.1063/1.1688752.
(26) Iikura, H.; Tsuneda, T.; Yanai, T.; Hirao, K. A Long-Range Correction Scheme for Generalized-Gradient-Approximation Exchange Functionals. *The Journal of Chemical Physics* **2001**, *115* (8), 3540–3544. https://doi.org/10.1063/1.1383587.
(27) Zhao, Y.; Truhlar, D. G. A New Local Density Functional for Main-Group Thermochemistry, Transition Metal Bonding, Thermochemical Kinetics, and Noncovalent Interactions. *The Journal of Chemical Physics* **2006**, *125* (19), 194101. https://doi.org/10.1063/1.2370993.
(28) Zhao, Y.; Truhlar, D. G. The M06 Suite of Density Functionals for Main Group Thermochemistry, Thermochemical Kinetics, Noncovalent Interactions, Excited States, and Transition Elements: Two New Functionals and Systematic Testing of Four M06-Class Functionals and 12 Other Functionals. *Theor Chem Account* **2008**, *120* (1–3), 215–241. https://doi.org/10.1007/s00214-007-0310-x.
(29) Cohen, A. J.; Handy, N. C. Dynamic Correlation. *Molecular Physics* **2001**, *99* (7), 607–615. https://doi.org/10.1080/00268970010023435.
(30) Hoe, W.-M.; Cohen, A. J.; Handy, N. C. Assessment of a New Local Exchange Functional OPTX. *Chemical Physics Letters* **2001**, *341* (3–4), 319–328. https://doi.org/10.1016/S0009-2614(01)00581-4.
(31) Perdew, J. P.; Tao, J.; Staroverov, V. N.; Scuseria, G. E. Meta-Generalized Gradient Approximation: Explanation of a Realistic

Nonempirical Density Functional. *The Journal of Chemical Physics* **2004**, *120* (15), 6898–6911. https://doi.org/10.1063/1.1665298.

(32) Tao, J.; Perdew, J. P.; Staroverov, V. N.; Scuseria, G. E. Climbing the Density Functional Ladder: Nonempirical Meta–Generalized Gradient Approximation Designed for Molecules and Solids. *Phys. Rev. Lett.* **2003**, *91* (14), 146401. https://doi.org/10.1103/PhysRevLett.91.146401.

(33) Staroverov, V. N.; Scuseria, G. E.; Tao, J.; Perdew, J. P. Comparative Assessment of a New Nonempirical Density Functional: Molecules and Hydrogen-Bonded Complexes. *The Journal of Chemical Physics* **2003**, *119* (23), 12129–12137. https://doi.org/10.1063/1.1626543.

(34) Chai, J.-D.; Head-Gordon, M. Systematic Optimization of Long-Range Corrected Hybrid Density Functionals. *The Journal of Chemical Physics* **2008**, *128* (8), 084106. https://doi.org/10.1063/1.2834918.

(35) Xu, X.; Goddard, W. A. The X3LYP Extended Density Functional for Accurate Descriptions of Nonbond Interactions, Spin States, and Thermochemical Properties. *Proc. Natl. Acad. Sci. U.S.A.* **2004**, *101* (9), 2673–2677. https://doi.org/10.1073/pnas.0308730100.

(36) Liu, Y.; Zhao, J.; Xu, X.; Xu, Y.; Cui, W.; Yang, Y.; Li, J. Emodin-Based Nanoarchitectonics with Giant Two-Photon Absorption for Enhanced Photodynamic Therapy. *Angewandte Chemie* **2023**, *135* (33), e202308019. https://doi.org/10.1002/ange.202308019.

(37) Guner, V. A.; Khuong, K. S.; Houk, K. N.; Chuma, A.; Pulay, P. The Performance of the Handy/Cohen Functionals, OLYP and O3LYP, for the Computation of Hydrocarbon Pericyclic Reaction Activation Barriers. *J. Phys. Chem. A* **2004**, *108* (15), 2959–2965. https://doi.org/10.1021/jp0369286.

7. XYZ-COORDINATES AND ENERGIES

```
==> -1 <==
29

O         1.596021          2.828779         -0.009384
O        -1.104017          2.414578         -0.007883
O        -0.191824         -2.890192         -0.021535
O        -3.508717          2.043824         -0.007343
O         5.565797         -0.168304         -0.030998
C         0.731284         -0.718076         -0.019837
C         0.511454          0.685307         -0.015177
C        -1.948156          0.244914         -0.013911
C        -1.751343         -1.140148         -0.018087
C        -0.827231          1.183856         -0.011720
C        -0.386147         -1.678072         -0.020514
C         1.997162         -1.245915         -0.022605
C         1.656963          1.569074         -0.013905
C         3.123460         -0.410227         -0.024297
C         2.945952          0.940339         -0.019240
C        -3.267355          0.739303         -0.011507
C        -2.826847         -2.010839         -0.019881
C         4.476894         -1.053861         -0.029422
C        -4.341193         -0.149539         -0.013505
C        -4.118354         -1.509029         -0.017656
H         2.120583         -2.321451         -0.023534
H         3.801050          1.606868         -0.022145
H        -2.648670         -3.077592         -0.023097
H         4.537718         -1.747589          0.819367
H         4.572499         -1.663498         -0.933059
H        -5.347115          0.254081         -0.011702
H        -4.962434         -2.189086         -0.019200
H        -2.564914          2.455215         -0.006664
H         5.593709          0.292285          0.816075

# ENERGIES
FINAL SINGLE POINT ENERGY: -951.910772257475
FINAL GIBBS FREE ENERGY: -951.74502014

==> -1/AEA <==
29

O         1.582035          2.834734         -0.017962
O        -1.129873          2.440198         -0.015504
O        -0.182915         -2.929209         -0.007549
O        -3.477561          2.055229         -0.015170
O         5.557389         -0.152517          0.027697
C         0.709805         -0.733313         -0.015347
C         0.495310          0.681917         -0.016745
C        -1.935162          0.247923         -0.013081
C        -1.726686         -1.149679         -0.011241
C        -0.839623          1.177479         -0.014627
C        -0.388912         -1.679058         -0.010573
C         2.001099         -1.252655         -0.016738
C         1.652643          1.562851         -0.020337
C         3.109245         -0.419982         -0.028661
C         2.930520          0.945232         -0.029845
C        -3.267492          0.743625         -0.013763
C        -2.830793         -2.013059         -0.010547
C         4.473167         -1.049147         -0.048264
C        -4.338840         -0.135735         -0.013233
C        -4.110068         -1.508699         -0.011740
H         2.129750         -2.327439         -0.009922
H         3.786782          1.610421         -0.040203
H        -2.662975         -3.081845         -0.009431
H         4.534264         -1.792886          0.756465
H         4.603203         -1.595977         -0.986911
H        -5.346137          0.264971         -0.014132
H        -4.957190         -2.186663         -0.011570
H        -2.461613          2.434328         -0.015378
H         5.526857          0.293958          0.881944

# ENERGIES
FINAL SINGLE POINT ENERGY: -952.033538275418
FINAL GIBBS FREE ENERGY: -951.87172853

==> -1/AIP <==
29

O         1.591011          2.791781         -0.006873
O        -1.096232          2.410565         -0.012331
O        -0.171240         -2.868630         -0.025241
O        -3.542348          2.037608         -0.011105
O         5.581164         -0.173736         -0.045752
C         0.722986         -0.697566         -0.021003
C         0.504797          0.669861         -0.015811
C        -1.965655          0.251415         -0.015892
C        -1.760107         -1.136372         -0.018936
C        -0.863061          1.190744         -0.014597
C        -0.396763         -1.669842         -0.022487
C         2.014070         -1.222866         -0.023396
C         1.672342          1.553528         -0.011609
C         3.152216         -0.408069         -0.022793
C         2.977072          0.941715         -0.014337
C        -3.287302          0.741594         -0.013485
C        -2.829373         -2.007182         -0.018822
C         4.499792         -1.060274         -0.028837
C        -4.358917         -0.154032         -0.013441
C        -4.126886         -1.508002         -0.015901
H         2.123443         -2.300943         -0.025347
H         3.821524          1.620061         -0.012265
H        -2.650353         -3.073804         -0.020974
H         4.556538         -1.745636          0.826552
H         4.579204         -1.678841         -0.927960
H        -5.366273          0.244867         -0.011383
H        -4.965641         -2.194108         -0.015663
H        -2.636344          2.482811         -0.011397
H         5.656566          0.248357          0.818721

# ENERGIES
FINAL SINGLE POINT ENERGY: -951.718953763389
FINAL GIBBS FREE ENERGY: -951.55399565

==> -1/UVVIS/S1 <==
29

O         1.310443          2.778384         -0.357490
O        -0.950218          2.403704         -0.247784
O        -0.152463         -2.952365          0.157301
O        -3.440880          2.065578         -0.124361
O         5.192867         -0.548332          1.073296
C         0.763779         -0.788802         -0.053520
C         0.514515          0.589552         -0.151527
C        -1.895383          0.246425         -0.032642
```

```
C         -1.680458         -1.149337          0.072431
C         -0.787934          1.143513         -0.148850
C         -0.344049         -1.714365          0.064355
C          2.086290         -1.231329         -0.070981
C          1.575402          1.529726         -0.266864
C          3.124707         -0.327088         -0.183772
C          2.887113          1.041686         -0.283612
C         -3.213592          0.753395         -0.024627
C         -2.788192         -1.991562          0.184188
C          4.542767         -0.813485         -0.162481
C         -4.289800         -0.107715          0.087286
C         -4.065195         -1.475156          0.191221
H          2.294434         -2.290932          0.003370
H          3.706678          1.743127         -0.376448
H         -2.627023         -3.058358          0.264996
H          4.566388         -1.885452         -0.382743
H          5.132368         -0.296787         -0.922373
H         -5.293280          0.300345          0.092162
H         -4.914541         -2.143486          0.278773
H         -2.550441          2.506897         -0.193730
H          4.731925         -1.042778          1.762062

# ENERGIES
FINAL SINGLE POINT ENERGY: -951.846128150851
FINAL GIBBS FREE ENERGY: -951.68127729

==> -1/UVVIS/S2 <==
29

O          1.570042          2.803788         -0.012595
O         -1.109662          2.421387         -0.009901
O         -0.163619         -2.906821         -0.022694
O         -3.509126          2.045322         -0.009689
O          5.575982         -0.166605         -0.020131
C          0.720592         -0.724408         -0.018979
C          0.507194          0.663051         -0.015981
C         -1.950709          0.252989         -0.014815
C         -1.733619         -1.149839         -0.017664
C         -0.869260          1.184705         -0.012897
C         -0.400912         -1.676901         -0.019942
C          2.012416         -1.238418         -0.020171
C          1.673169          1.551278         -0.015394
C          3.134023         -0.408550         -0.024564
C          2.965261          0.949989         -0.019954
C         -3.283184          0.742866         -0.012467
C         -2.833841         -2.006587         -0.018077
C          4.487740         -1.052778         -0.033516
C         -4.360400         -0.137479         -0.012941
C         -4.121399         -1.501965         -0.015821
H          2.136634         -2.314216         -0.017973
H          3.813946          1.622881         -0.024812
H         -2.669040         -3.075902         -0.020026
H          4.546130         -1.757306          0.806288
H          4.586468         -1.649988         -0.944992
H         -5.366551          0.263298         -0.011230
H         -4.962180         -2.186984         -0.016552
H         -2.563923          2.458845         -0.009586
H          5.604058          0.279355          0.834709

# ENERGIES
FINAL SINGLE POINT ENERGY: -951.836480684090
FINAL GIBBS FREE ENERGY: -951.67413649

==> -1/UVVIS/T1 <==
29

O          1.584309          2.792208         -0.009749
O         -1.118450          2.440763         -0.012490
O         -0.161883         -2.902282         -0.016763
O         -3.514867          2.050609         -0.012572
O          5.573592         -0.165357         -0.021938
C          0.695145         -0.710233         -0.016318
C          0.482251          0.671114         -0.014510
C         -1.951881          0.256352         -0.014029
C         -1.735209         -1.140591         -0.015303
C         -0.867979          1.192993         -0.013477
C         -0.393906         -1.664988         -0.016252
C          2.014283         -1.228991         -0.018178
C          1.662350          1.547253         -0.014468
C          3.137236         -0.418541         -0.025226
C          2.960802          0.942135         -0.021440
C         -3.286715          0.741862         -0.013792
C         -2.829325         -2.008075         -0.016185
C          4.493447         -1.060735         -0.037193
C         -4.352487         -0.142165         -0.014880
C         -4.113756         -1.510014         -0.016043
H          2.127402         -2.305820         -0.015568
H          3.804671          1.620650         -0.024955
H         -2.654859         -3.075565         -0.017237
H          4.556461         -1.766627          0.800257
H          4.593984         -1.651823         -0.951986
H         -5.361447          0.252847         -0.014834
H         -4.955447         -2.193873         -0.016923
H         -2.563978          2.459251         -0.011963
```

```
H          5.612482          0.262648          0.841649

# ENERGIES
FINAL SINGLE POINT ENERGY: -951.862174187001
FINAL GIBBS FREE ENERGY: -951.69790668

==> -1/UVVIS/T1/AEA <==
29

O          1.582364          2.834800         -0.018767
O         -1.129662          2.440092         -0.016296
O         -0.182843         -2.929268         -0.007581
O         -3.477486          2.055103         -0.015024
O          5.557474         -0.152829          0.031440
C          0.709968         -0.733444         -0.015838
C          0.495621          0.681857         -0.017369
C         -1.934943          0.247877         -0.013278
C         -1.726563         -1.149727         -0.011341
C         -0.839335          1.177538         -0.015276
C         -0.388830         -1.679117         -0.011154
C          2.001198         -1.252848         -0.016978
C          1.652898          1.562840         -0.020986
C          3.109311         -0.420118         -0.028946
C          2.930663          0.945094         -0.030407
C         -3.267298          0.743457         -0.013588
C         -2.830773         -2.012991         -0.010141
C          4.473285         -1.049191         -0.048639
C         -4.338771         -0.135725         -0.012568
C         -4.110043         -1.508717         -0.010949
H          2.130551         -2.327541         -0.009908
H          3.786782          1.610495         -0.040886
H         -2.663162         -3.081818         -0.008960
H          4.533288         -1.795355          0.753932
H          4.604638         -1.593113         -0.988799
H         -5.345877          0.265442         -0.013150
H         -4.957063         -2.186803         -0.010386
H         -2.462229          2.434456         -0.015595
H          5.523064          0.294560          0.885076

# ENERGIES
FINAL SINGLE POINT ENERGY: -952.033538996802
FINAL GIBBS FREE ENERGY: -951.87180996

==> -1/UVVIS/T1/AIP <==
29

O          1.590908          2.791902         -0.005274
O         -1.096286          2.410558         -0.012440
O         -0.171170         -2.868544         -0.024039
O         -3.542234          2.037562         -0.011583
O          5.581221         -0.174483         -0.047095
C          0.722971         -0.697508         -0.020599
C          0.504763          0.669931         -0.015116
C         -1.965572          0.251429         -0.015570
C         -1.760050         -1.136374         -0.018521
C         -0.863060          1.190718         -0.013854
C         -0.396807         -1.669765         -0.021624
C          2.013972         -1.222718         -0.023559
C          1.672334          1.553692         -0.010979
C          3.152167         -0.407832         -0.022910
C          2.977046          0.941922         -0.014098
C         -3.287286          0.741541         -0.013708
C         -2.829336         -2.007208         -0.018779
C          4.499431         -1.060544         -0.029075
C         -4.358863         -0.154082         -0.014002
C         -4.126864         -1.508019         -0.016344
H          2.124341         -2.300682         -0.026021
H          3.821584          1.620173         -0.011987
H         -2.650448         -3.073850         -0.020901
H          4.556192         -1.745157          0.826930
H          4.578273         -1.679968         -0.927638
H         -5.366135          0.245062         -0.012330
H         -4.965607         -2.194150         -0.016410
H         -2.636021          2.482692         -0.011667
H          5.656762          0.248706          0.816828

# ENERGIES
FINAL SINGLE POINT ENERGY: -951.718953758747
FINAL GIBBS FREE ENERGY: -951.55400996

==> -1/UVVIS/T2 <==
29

O          1.340947          2.716674         -0.044152
O         -0.913137          2.387493         -0.014865
O         -0.238302         -3.003731         -0.005281
O         -3.426572          2.091236         -0.017924
O          5.526880         -0.033285          0.062198
C          0.727091         -0.850131         -0.019741
C          0.501969          0.536839         -0.024324
C         -1.912144          0.239518         -0.013887
C         -1.726113         -1.165262         -0.009861
C         -0.783325          1.115291         -0.016557
C         -0.402434         -1.758413         -0.010448
```

```
C          2.038627          -1.312631          -0.020874
C          1.581047           1.460400          -0.033723
C          3.099136          -0.421457          -0.031602
C          2.886346           0.950648          -0.033573
C         -3.222240           0.769145          -0.013394
C         -2.853183          -1.991778          -0.004711
C          4.495604          -0.980209          -0.057978
C         -4.314509          -0.075527          -0.008557
C         -4.119080          -1.453330          -0.004262
H          2.224191          -2.379767          -0.010993
H          3.717079           1.642891          -0.043989
H         -2.712142          -3.064535          -0.001169
H          4.582175          -1.749777           0.718451
H          4.655348          -1.482968          -1.016459
H         -5.310323           0.351153          -0.008557
H         -4.983195          -2.108225          -0.000668
H         -2.534270           2.524686          -0.020901
H          5.510760           0.330060           0.955442

# ENERGIES
FINAL SINGLE POINT ENERGY: -951.850478882507
FINAL GIBBS FREE ENERGY: -951.68561317

==> -1/UVVIS/T3 <==
29

O          1.558423           2.828717          -0.011996
O         -1.116632           2.434473          -0.015068
O         -0.208310          -2.899021          -0.006411
O         -3.519185           2.015003          -0.017363
O          5.547654          -0.137544           0.006785
C          0.704297          -0.721935          -0.012880
C          0.489736           0.681254          -0.014068
C         -1.949601           0.246732          -0.013102
C         -1.740906          -1.129451          -0.011165
C         -0.840520           1.153384          -0.013531
C         -0.401539          -1.656408          -0.009559
C          2.000332          -1.248259          -0.014410
C          1.643733           1.568833          -0.016838
C          3.105853          -0.416964          -0.026560
C          2.924572           0.951221          -0.026690
C         -3.300223           0.757168          -0.015503
C         -2.850201          -2.018937          -0.011808
C          4.471802          -1.040244          -0.047305
C         -4.370922          -0.166990          -0.016242
C         -4.134565          -1.533377          -0.014530
H          2.123912          -2.323162          -0.008682
H          3.779864           1.616386          -0.035165
H         -2.658301          -3.082895          -0.010375
H          4.538101          -1.773814           0.765803
H          4.589841          -1.598835          -0.980479
H         -5.378784           0.230870          -0.018603
H         -4.971955          -2.221613          -0.015449
H         -2.157699           2.488192          -0.016188
H          5.557452           0.282221           0.875015

# ENERGIES
FINAL SINGLE POINT ENERGY: -951.840567669584
FINAL GIBBS FREE ENERGY: -951.67723619

==> 0 <==
30

O          1.495371           2.766836          -0.003390
O         -0.990813           2.377768           0.004045
O         -0.229472          -2.941570          -0.004265
O         -3.484539           2.060452           0.009303
O          5.548797          -0.124892          -0.042628
C          0.739407          -0.798496          -0.007826
C          0.520687           0.590753          -0.004369
C         -1.943005           0.236496           0.003065
C         -1.758988          -1.156488           0.000868
C         -0.810527           1.131435           0.001249
C         -0.401881          -1.731335          -0.003891
C          2.018493          -1.300320          -0.014033
C          1.633486           1.448514          -0.007234
C          3.120489          -0.441751          -0.020327
C          2.922872           0.919157          -0.015589
C         -3.250221           0.755334           0.007152
C         -2.848354          -2.002366           0.002962
C          4.491879          -1.044291          -0.034471
C         -4.339114          -0.115994           0.009034
C         -4.134031          -1.475530           0.007040
H          2.168704          -2.372698          -0.014523
H          3.761681           1.603929          -0.021287
H         -2.692996          -3.072656           0.001315
H          4.576585          -1.737514           0.811997
H          4.594151          -1.649214          -0.940561
H         -5.338518           0.302495           0.012118
H         -4.987600          -2.143279           0.008626
H         -2.590424           2.502758           0.007758
H          5.608353           0.292376           0.825076
H          0.513441           2.944263           0.000774
```

```
# ENERGIES
FINAL SINGLE POINT ENERGY: -952.381301554829
FINAL GIBBS FREE ENERGY: -952.20130721

==> 0/AEA <==
30

O          1.436226           2.822046          -0.275681
O         -1.010677           2.427995          -0.170497
O         -0.155854          -2.940564           0.108139
O         -3.459889           2.058371          -0.049277
O          5.275285          -0.508463           0.986455
C          0.749262          -0.767151          -0.047250
C          0.515299           0.626102          -0.116636
C         -1.905600           0.247309          -0.004018
C         -1.693677          -1.150699           0.070690
C         -0.807534           1.149623          -0.100434
C         -0.353179          -1.700828           0.047586
C          2.063469          -1.241119          -0.066692
C          1.627963           1.501479          -0.205950
C          3.133219          -0.377792          -0.157401
C          2.910859           0.999455          -0.228014
C         -3.232868           0.744114           0.020475
C         -2.794971          -2.004633           0.167704
C          4.534192          -0.902380          -0.161146
C         -4.301597          -0.125760           0.117576
C         -4.073711          -1.496049           0.190706
H          2.236997          -2.308202          -0.009527
H          3.743167           1.690483          -0.302784
H         -2.628489          -3.071978           0.225251
H          4.518912          -1.993308          -0.251145
H          5.085860          -0.502041          -1.015301
H         -5.307548           0.277009           0.135746
H         -4.920403          -2.169259           0.267057
H         -2.542412           2.479061          -0.112211
H          4.838993          -0.880230           1.762802
H          0.432620           2.941581          -0.248237

# ENERGIES
FINAL SINGLE POINT ENERGY: -952.519914478140
FINAL GIBBS FREE ENERGY: -952.34295582

==> 0/AIP <==
30

O          1.466214           2.729488           0.000208
O         -0.982099           2.370819           0.002847
O         -0.223035          -2.947162          -0.013694
O         -3.464526           2.044274           0.009068
O          5.563804          -0.131638          -0.059515
C          0.732053          -0.799573          -0.010183
C          0.511652           0.569472          -0.003966
C         -1.935422           0.227808           0.001434
C         -1.758558          -1.160273          -0.002015
C         -0.817307           1.123595           0.000358
C         -0.405327          -1.744674          -0.008932
C          2.029390          -1.283165          -0.015997
C          1.642779           1.448786          -0.003343
C          3.144569          -0.416757          -0.018798
C          2.949748           0.934376          -0.009207
C         -3.255327           0.758022           0.006840
C         -2.860045          -1.992052           0.001120
C          4.502206          -1.033876          -0.029789
C         -4.360020          -0.108811           0.010183
C         -4.154672          -1.459772           0.007520
H          2.188140          -2.355223          -0.017997
H          3.778700           1.630356          -0.010570
H         -2.714758          -3.064292          -0.001257
H          4.571241          -1.716752           0.828782
H          4.581091          -1.663403          -0.922978
H         -5.354535           0.319544           0.014715
H         -5.003800          -2.132246           0.010184
H         -2.566523           2.495736           0.006366
H          5.650201           0.288616           0.805209
H          0.458078           2.892949           0.001393

# ENERGIES
FINAL SINGLE POINT ENERGY: -952.152620800187
FINAL GIBBS FREE ENERGY: -951.97579692

==> 0/MOLECULARDYNAMICS/AT-TA/NCI <==
286

P         23.373000          20.053000          38.053000
O         22.517000          19.151000          38.927000
O         23.345000          21.481000          38.351000
O         24.854000          19.639000          38.141000
C         25.505000          18.456000          37.643000
H         24.727000          17.991000          37.037000
H         25.879000          17.779000          38.411000
C         26.689000          18.843000          36.819000
H         27.224000          17.899000          36.713000
O         26.233000          19.306000          35.578000
C         26.916000          20.498000          35.248000
```

H	27.877000	20.295000	34.775000
N	26.097000	21.361000	34.334000
C	25.089000	22.185000	34.687000
H	24.641000	22.192000	35.670000
N	24.680000	22.981000	33.713000
C	25.475000	22.583000	32.616000
C	25.654000	23.048000	31.241000
N	24.870999	23.924000	30.561000
H	25.322000	24.301000	29.739000
H	24.190000	24.408000	31.128000
N	26.523000	22.387000	30.515000
C	27.291000	21.463000	31.047000
H	27.847000	20.880000	30.327000
N	27.231000	20.934000	32.238000
C	26.277000	21.560000	32.976000
C	27.598000	19.876000	37.531000
H	27.284000	20.061000	38.558000
C	27.230000	21.056000	36.618000
H	26.279000	21.449000	36.978000
H	27.971000	21.853000	36.551000
O	28.955000	19.321000	37.640000
P	30.166364	20.370923	38.047984
O	31.353518	19.484791	38.318088
O	29.660384	21.371567	39.054403
O	30.409801	21.247417	36.679256
C	30.998527	20.593682	35.540667
H	30.410018	19.708529	35.262568
H	32.022935	20.273441	35.771395
C	31.058589	21.538614	34.370061
H	31.572924	21.008874	33.552415
O	29.720188	21.891805	33.945236
C	29.732053	23.167761	33.331262
H	29.356199	23.110629	32.303184
N	28.750984	24.020336	34.052599
C	28.481282	24.018558	35.375396
H	29.065467	23.435320	36.072824
N	27.423756	24.790781	35.721379
C	27.040322	25.347322	34.568285
C	25.978540	26.238874	34.215070
N	25.121196	26.709433	35.094492
H	24.394407	27.352411	34.794358
H	25.202060	26.478388	36.078842
N	25.842981	26.605897	32.906287
C	26.697108	26.131846	32.025067
H	26.568842	26.472800	31.000574
N	27.747080	25.266142	32.229556
C	27.849326	24.894004	33.476002
C	31.739031	22.888714	34.581587
H	31.452148	23.315299	35.550424
C	31.160076	23.693531	33.415082
H	31.199058	24.777436	33.533081
H	31.684680	23.412441	32.496062
O	33.155360	22.708809	34.520585
P	34.129282	23.880891	35.149652
O	35.527876	23.355738	34.961227
O	33.624647	24.325444	36.497573
O	33.842166	25.153237	34.158091
C	34.281880	25.107195	32.787616
H	33.430567	24.872676	32.138359
H	35.059609	24.346176	32.647018
C	34.859337	26.445822	32.414498
H	35.295598	26.371921	31.410009
O	33.803973	27.444243	32.391304
C	34.341534	28.625461	32.991666
H	34.941382	29.178666	32.259232
N	33.218710	29.502737	33.336732
C	32.632323	29.505918	34.551403
H	33.095291	28.900428	35.323090
C	31.510685	30.255667	34.840108
C	30.931684	30.330636	36.192454
H	29.862464	30.069038	36.169768
H	31.455685	29.681123	36.895037
H	30.964657	31.366886	36.556671
C	30.908862	31.031330	33.767460
O	29.861601	31.677671	33.889255
N	31.581427	30.997112	32.564650
H	31.112777	31.451345	31.753836
C	32.665656	30.225106	32.255718
O	33.133741	30.158203	31.129758
C	35.921777	26.972284	33.404615
H	36.257147	26.191683	34.092821
C	35.213341	28.114659	34.121147
H	34.614026	27.692367	34.932237
H	35.908870	28.858599	34.511692
O	37.036931	27.522275	32.676392
P	38.418000	26.658000	32.511000
O	38.168000	25.194000	32.617000
O	39.464000	27.209000	33.352000
O	38.816000	27.027000	30.986000
C	37.928000	26.713000	29.914000
H	37.110000	26.043000	30.181000
H	38.496000	26.128000	29.191000
C	37.431000	27.879000	29.141000
H	36.974000	27.451000	28.249000

O	36.520000	28.710000	29.759000
C	36.840000	30.072000	29.414000
H	36.461000	30.230000	28.405000
N	36.387000	31.027000	30.473000
C	36.943000	30.963000	31.729000
H	37.836000	30.381000	31.903000
C	36.351000	31.698000	32.737000
C	36.916000	31.663000	34.146000
H	37.076000	32.636000	34.611000
H	36.276000	31.110000	34.833000
H	37.893000	31.179000	34.174000
C	35.231000	32.603000	32.423000
O	34.631000	33.188000	33.261000
N	34.891000	32.668000	31.065000
H	34.017000	33.126000	30.850000
C	35.376000	31.888000	30.117000
O	34.793000	31.943000	29.002000
C	38.562000	28.776000	28.550000
H	39.555000	28.357000	28.712000
C	38.304000	30.109000	29.203000
H	38.827000	30.110000	30.159000
H	38.598000	30.994000	28.639000
O	38.576000	28.740000	27.147000
P	26.046000	40.504000	29.998000
O	25.032000	41.181000	29.100000
O	25.562000	39.679000	31.138000
O	26.922000	39.627000	29.021000
C	27.517000	40.310000	27.986000
H	28.099000	41.107000	28.449000
H	26.710000	40.797000	27.438000
C	28.393000	39.358000	27.076000
H	28.892000	39.973000	26.327000
O	29.371000	38.573000	27.875000
C	29.207000	37.192000	27.651000
H	29.826000	36.833000	26.829000
N	29.483000	36.467000	28.924000
C	28.946000	36.814000	30.125000
H	28.107000	37.491000	30.188000
N	29.542000	36.176000	31.120000
C	30.650000	35.477000	30.503000
C	31.732000	34.736000	31.071000
N	32.046000	34.723000	32.322000
H	33.015000	34.564000	32.558000
H	31.491000	35.161000	33.044000
N	32.564000	34.092000	30.170000
C	32.486000	34.311000	28.895000
H	32.978000	33.618000	28.229000
N	31.477000	35.020000	28.278000
C	30.587000	35.598000	29.138000
C	27.525000	38.320000	26.331000
H	26.463000	38.566000	26.345000
C	27.803000	37.078000	27.156000
H	27.123000	36.905000	27.990000
H	27.621000	36.148000	26.619000
O	27.862000	38.134000	24.957000
P	26.843641	37.325279	23.939611
O	27.063577	37.953731	22.588225
O	25.476341	37.202021	24.557233
O	27.460866	35.805230	23.942364
C	28.764862	35.618706	23.360439
H	29.474226	36.342716	23.783064
H	28.722479	35.758045	22.272729
C	29.268678	34.227969	23.654427
H	30.256429	34.138550	23.185347
O	29.419486	34.063462	25.093074
C	28.742519	32.872261	25.481850
H	29.439967	32.025908	25.491225
N	28.306349	33.008896	26.868316
C	27.148936	33.462840	27.402439
H	26.327344	33.827419	26.798616
N	27.116593	33.401569	28.751018
C	28.313733	32.890438	29.084842
C	28.903002	32.499668	30.326450
N	28.287198	32.572719	31.484019
H	28.768782	32.257789	32.336822
H	27.340978	32.936155	31.539741
N	30.169601	31.980141	30.289418
C	30.785260	31.827316	29.135147
H	31.785512	31.399794	29.167561
N	30.304385	32.121286	27.892572
C	29.098462	32.629093	27.934690
C	28.385699	33.053925	23.187424
H	27.707926	33.333224	22.374852
C	27.648102	32.656006	24.460371
H	26.823271	33.358904	24.610356
H	27.268152	31.633908	24.455885
O	29.315943	32.050813	22.713264
P	28.973199	30.466631	22.441882
O	29.865878	30.068687	21.293108
O	27.489745	30.210028	22.408078
O	29.514964	29.747987	23.821562
C	30.904488	29.909977	24.175336
H	31.001414	30.730950	24.895020
H	31.513760	30.147507	23.293996

```
C         31.443553          28.636508          24.778153
H         32.496909          28.809817          25.038432
O         30.710996          28.323355          25.990652
C         30.457821          26.925172          26.022272
H         31.253982          26.392174          26.553462
N         29.215595          26.702945          26.772565
C         28.064503          27.256263          26.314081
H         28.158182          27.906192          25.448907
C         26.845767          27.025120          26.889398
C         25.587906          27.590627          26.353016
H         24.931700          26.784021          25.997633
H         25.033460          28.111040          27.144450
H         25.785456          28.278989          25.529350
C         26.793283          26.140559          28.034696
O         25.740584          25.784113          28.571024
N         28.017756          25.696534          28.504672
H         28.015608          25.106082          29.333873
C         29.253690          25.957749          27.964652
O         30.292710          25.573815          28.479737
C         31.348002          27.400557          23.863682
H         31.010529          27.672859          22.859250
C         30.345585          26.489258          24.562575
H         29.344102          26.698171          24.174650
H         30.553769          25.426071          24.433875
O         32.671652          26.824267          23.773213
P         32.880000          25.525000          22.790000
O         34.325000          25.546000          22.340000
O         31.798000          25.464000          21.784000
O         32.653000          24.253000          23.796000
C         33.370000          24.194000          25.007000
H         33.277000          25.108000          25.594000
H         34.430000          24.096000          24.775000
C         32.853000          23.064000          25.978000
H         33.463000          23.019000          26.880000
O         31.524000          23.450000          26.228000
C         30.789000          22.200000          26.347000
H         31.024000          21.706000          27.290000
N         29.309000          22.489000          26.402000
C         28.617000          22.974000          25.313000
H         29.128000          23.093000          24.369000
C         27.290000          23.276000          25.345000
C         26.561000          23.952000          24.115000
H         25.721000          23.355000          23.759000
H         26.088000          24.877000          24.445000
H         27.343000          24.180000          23.391000
C         26.511000          23.009000          26.513000
O         25.281000          23.195000          26.748000
N         27.282000          22.584000          27.576000
H         26.780000          22.280000          28.398000
C         28.642000          22.329000          27.646000
O         29.239000          22.045000          28.663000
C         32.818000          21.614000          25.472000
H         33.419000          21.547000          24.566000
C         31.271000          21.334000          25.196000
H         31.012578          21.719998          24.206100
H         31.023682          20.279482          25.309113
O         33.258360          20.746578          26.398933
O         26.784503          28.950376          35.631343
O         26.010198          29.970779          33.486938
O         30.495598          28.330346          31.015722
O         24.928495          30.534816          31.155410
O         30.470012          25.573008          36.678582
C         29.207966          28.363098          32.995551
C         28.034287          28.799080          33.596296
C         27.109744          29.609099          31.402302
C         28.292294          29.166067          30.768183
C         26.980979          29.506877          32.855939
C         29.424240          28.604221          31.547543
C         30.193057          27.696161          33.749248
C         27.850741          28.528471          34.992062
C         29.979693          27.374350          35.100556
C         28.813023          27.784910          35.712303
C         26.058380          30.119633          30.581648
C         28.437013          29.263104          29.387893
C         31.026141          26.613911          35.878007
C         26.214071          30.177316          29.185982
C         27.393024          29.770728          28.602218
H         31.100642          27.367085          33.249865
H         28.602045          27.567276          36.755606
H         29.364268          28.938811          28.925855
H         31.738603          26.153403          35.189994
H         31.582337          27.325289          36.507278
H         25.397238          30.568002          28.585152
H         27.518081          29.846016          27.526755
H         26.237303          29.473137          34.944323
H         24.305297          30.846369          30.477086
H         30.090407          25.958471          37.480270

# ENERGIES
FINAL SINGLE POINT ENERGY: -11931.364869055962

==> 0/MOLECULARDYNAMICS/CG-GC/NCI <==
283
```

```
P         13.418000          15.455000          37.704000
O         13.126000          15.018000          39.044000
O         13.563000          16.911000          37.392000
O         14.687000          14.671000          37.192000
C         15.999000          15.139000          37.538000
H         16.105000          14.943000          38.605000
H         16.087000          16.162000          37.174000
C         17.188000          14.402000          36.841000
H         17.330000          13.441000          37.334000
O         17.052000          14.344000          35.382000
C         18.023000          15.077000          34.682000
H         18.833000          14.467000          34.283000
N         17.224000          15.770000          33.620000
C         16.301000          16.713000          33.662000
H         15.887000          16.958000          34.629000
N         16.040000          17.345000          32.513000
C         16.757000          16.543000          31.554000
C         16.843000          16.643000          30.108000
O         16.299000          17.396000          29.203000
N         17.728000          15.661000          29.663000
H         17.808000          15.591000          28.659000
C         18.625000          14.964000          30.407000
N         19.515000          14.248000          29.804000
H         19.499000          14.195000          28.796000
H         19.962000          13.535000          30.363000
N         18.610000          14.937000          31.753000
C         17.599000          15.648000          32.273000
C         18.457000          15.205000          37.097000
H         18.367000          15.887000          37.942000
C         18.534000          16.024000          35.747000
H         17.904000          16.912000          35.804000
H         19.567000          16.363000          35.674000
O         19.591000          14.402000          37.231000
P         20.948788          14.912860          38.027197
O         21.483011          13.694688          38.732560
O         20.665696          16.205884          38.742825
O         21.936444          15.299148          36.780265
C         22.478272          14.222329          35.988118
H         21.715782          13.455364          35.802660
H         23.324735          13.758068          36.509945
C         22.958786          14.778070          34.673725
H         23.411211          13.956591          34.095423
O         21.834331          15.322353          33.961044
C         22.264453          16.378902          33.096872
H         22.186814          16.055253          32.060052
N         21.308936          17.491496          33.253376
C         21.279879          18.207241          34.407149
H         22.018171          17.960555          35.163291
C         20.358431          19.184115          34.603564
H         20.347803          19.760968          35.520667
C         19.410482          19.414331          33.563114
N         18.468472          20.345707          33.714123
H         17.796092          20.512281          32.979359
H         18.414122          20.880526          34.567739
N         19.444000          18.724301          32.413789
C         20.368082          17.768296          32.240258
O         20.412933          17.088893          31.186248
C         23.963435          15.927188          34.764250
H         23.773853          16.535472          35.655229
C         23.714418          16.711085          33.476070
H         23.893868          17.779520          33.592117
H         24.377591          16.331679          32.693593
O         25.285552          15.372792          34.833296
P         26.492306          16.227073          35.524462
O         27.682724          15.304371          35.577049
O         26.022885          16.921647          36.781034
O         26.715006          17.353485          34.340838
C         27.733100          18.343228          34.590574
H         28.623377          17.873350          35.031063
H         27.347934          19.105853          35.280031
C         28.139831          18.968869          33.274767
H         28.487249          18.196664          32.578770
O         27.007945          19.659197          32.663907
C         27.390790          21.019747          32.458190
H         27.839806          21.141625          31.463866
N         26.190266          21.856153          32.432503
C         25.466360          22.395840          33.434591
H         25.708566          22.242923          34.479735
N         24.419834          23.150374          33.009907
C         24.464187          23.065143          31.683864
C         23.622519          23.660034          30.649838
O         22.650981          24.377077          30.840941
N         24.046197          23.309073          29.368586
H         23.461170          23.667932          28.565405
C         25.103580          22.494696          29.089242
N         25.397893          22.214144          27.844127
H         24.818632          22.586375          27.047564
H         26.179896          21.591457          27.670105
N         25.922749          21.947276          30.053902
C         25.568266          22.251835          31.261240
C         29.220355          20.044843          33.483009
H         29.797904          19.876134          34.397693
C         28.398303          21.325057          33.547263
H         27.908589          21.393531          34.523710
```

H	28.990345	22.220890	33.362916
O	30.075969	20.099667	32.322005
P	31.664000	19.691000	32.580000
O	31.683000	18.275000	33.000000
O	32.245000	20.703000	33.470000
O	32.279000	19.835000	31.159000
C	31.825000	18.978000	30.110000
H	30.913000	18.448000	30.385000
H	32.574000	18.226000	29.861000
C	31.406000	19.885000	28.947000
H	31.213000	19.238000	28.091000
O	30.366000	20.654000	29.399000
C	30.564000	22.019000	28.957000
H	30.291000	22.127000	27.908000
N	29.748000	22.949000	29.680000
C	29.792000	23.411000	30.981000
H	30.541000	23.029000	31.658000
N	28.854000	24.265000	31.307000
C	28.158000	24.461000	30.111000
C	27.117000	25.292000	29.662000
N	26.319000	26.058000	30.484000
H	25.599000	26.660000	30.110000
H	26.557000	26.082000	31.466000
N	26.758000	25.225000	28.432000
C	27.333000	24.415000	27.588000
H	26.942000	24.383000	26.582000
N	28.393000	23.686000	27.862000
C	28.800000	23.797000	29.117000
C	32.528000	20.782000	28.519000
H	33.573000	20.570000	28.746000
C	32.114000	22.137000	29.094000
H	32.298000	22.293000	30.156000
H	32.373000	23.047000	28.553000
O	32.360000	20.994000	27.142000
P	21.076000	32.680000	24.865000
O	20.193000	33.442000	23.944000
O	20.392000	32.133000	26.034000
O	21.857000	31.425000	24.159000
C	22.946000	31.708000	23.229000
H	23.548000	32.440000	23.767000
H	22.603000	32.167000	22.302000
C	23.755000	30.451000	22.898000
H	24.439000	30.760000	22.107000
O	24.448000	29.989000	24.021000
C	24.033000	28.635000	24.345000
H	24.828000	28.004000	23.949000
N	23.972000	28.212000	25.811000
C	22.868000	28.676000	26.534000
H	22.199000	29.433000	26.153000
C	22.826000	28.445000	27.883000
C	21.793000	29.038000	28.839000
H	21.423000	28.210000	29.445000
H	22.339000	29.621000	29.581000
H	21.010000	29.656000	28.399000
C	23.744000	27.511000	28.403000
O	23.781000	27.210000	29.571000
N	24.716000	26.980000	27.562000
H	25.352000	26.331000	28.001000
C	24.690000	27.137000	26.201000
O	25.507000	26.512000	25.518000
C	22.887000	29.366000	22.387000
H	21.869000	29.696000	22.184000
C	22.886000	28.282000	23.523000
H	21.922000	28.368000	24.025000
H	23.050000	27.258000	23.187000
O	23.513000	28.975000	21.174000
P	22.695097	28.028296	20.096644
O	23.525057	28.081513	18.840832
O	21.230042	28.376490	20.109519
O	22.786556	26.535626	20.768339
C	24.072494	25.885598	20.784582
H	24.823305	26.528058	21.264464
H	24.395795	25.666249	19.759362
C	23.958956	24.589153	21.543956
H	24.885146	24.020096	21.388646
O	23.804363	24.866398	22.957544
C	22.839096	23.937372	23.466456
H	23.314292	22.971475	23.670207
N	22.362209	24.431352	24.756388
C	21.418649	25.403503	24.856355
H	21.019651	25.803746	23.932047
C	20.988116	25.840007	26.068935
H	20.225573	26.605415	26.146362
C	21.542280	25.221618	27.230239
N	21.080277	25.524751	28.440483
H	21.513566	25.125257	29.268695
H	20.375115	26.240027	28.542214
N	22.539629	24.326386	27.131693
C	22.997636	23.948872	25.923101
O	23.966921	23.173746	25.787811
C	22.753350	23.711153	21.138203
H	22.276289	24.070381	20.221224
C	21.820949	23.789267	22.349243
H	21.185886	24.673779	22.250234

H	21.200688	22.896906	22.454150
O	23.253467	22.371471	20.953585
P	22.430233	21.308981	20.005780
O	23.027335	21.294365	18.621877
O	20.943603	21.511432	20.179584
O	22.933210	19.930831	20.721155
C	22.494091	19.670576	22.069217
H	21.397492	19.638272	22.114282
H	22.856392	20.454482	22.744471
C	23.101695	18.339315	22.479775
H	24.194684	18.403692	22.472496
O	22.691990	18.025381	23.835862
C	21.631951	17.072652	23.794836
H	21.950364	16.156592	24.303561
N	20.520470	17.602209	24.611048
C	19.467629	18.366784	24.274225
H	19.206243	18.609141	23.251868
N	18.748869	18.821170	25.345429
C	19.380452	18.331752	26.396018
C	19.155310	18.518938	27.824561
O	18.319055	19.244098	28.334472
N	20.063748	17.777409	28.582913
H	19.956307	17.779723	29.617488
C	21.081428	17.022751	28.082743
N	21.873431	16.374253	28.901830
H	21.721803	16.426786	29.909781
H	22.645046	15.839931	28.518878
N	21.358480	16.903728	26.740356
C	20.516185	17.535621	25.991934
C	22.612433	17.159274	21.656315
H	22.489053	17.407367	20.598477
C	21.284123	16.825022	22.335918
H	20.518563	17.509390	21.963312
H	20.945267	15.801292	22.169267
O	23.581674	16.113232	21.823383
P	23.346000	14.701000	20.969000
O	24.739000	14.160000	20.770000
O	22.501000	14.989000	19.891000
O	22.592000	13.810000	22.060000
C	23.304000	13.038000	23.025000
H	24.071000	13.626000	23.529000
H	23.768000	12.169000	22.559000
C	22.298000	12.476000	23.999000
H	22.839000	11.890000	24.741000
O	21.472000	13.509000	24.603000
C	20.106000	13.023000	24.574000
H	20.013000	12.245000	25.332000
N	19.106000	13.977000	24.871000
C	18.313000	14.526000	23.872000
H	18.348000	14.226000	22.835000
C	17.264000	15.403000	24.251000
H	16.563000	15.658000	23.470000
C	17.125000	15.615000	25.654000
N	16.208000	16.432000	26.077000
H	16.109000	16.599000	27.068000
H	15.598000	16.762000	25.343000
N	17.926000	15.205000	26.593000
C	18.965000	14.408000	26.185000
O	19.736000	13.963000	27.027000
C	21.305000	11.584000	23.267000
H	21.633000	11.489000	22.232000
C	20.052000	12.391000	23.178000
H	20.079000	13.127000	22.374000
H	19.108000	11.857000	23.072000
O	21.143000	10.405000	23.908000
O	21.473462	20.870547	24.912842
O	22.976156	19.833261	26.744603
O	20.079428	22.312067	30.571884
O	24.395271	18.670670	28.541937
O	16.675435	23.414856	26.784896
C	20.393069	21.889159	28.266453
C	21.164332	21.243324	27.277058
C	22.592758	20.190258	29.047874
C	21.864159	20.854281	30.052073
C	22.284032	20.392884	27.638009
C	20.722753	21.730162	29.700300
C	19.336103	22.708255	27.908084
C	20.825701	21.459169	25.910473
C	19.037017	22.947851	26.549920
C	19.773428	22.324802	25.566485
C	23.661051	19.332563	29.434972
C	22.218302	20.711754	31.391542
C	17.903397	23.875471	26.207117
C	23.978433	19.165411	30.794725
C	23.275914	19.866345	31.751229
H	18.750724	23.193081	28.682492
H	19.559991	22.483664	24.512962
H	21.653677	21.241073	32.152048
H	18.092732	24.863279	26.641495
H	17.816768	23.984820	25.118666
H	24.796650	18.503280	31.059086
H	23.544630	19.776999	32.799368
H	22.199627	20.314479	25.305020
H	24.061627	18.920552	27.638267

```
H         16.461828         22.563347         26.377722

# ENERGIES
FINAL SINGLE POINT ENERGY: -11979.579888889817

==> 0/MOLECULARDYNAMICS/TC-AG/NCI <==
284

P         21.511000         13.774001         37.985999
O         22.321000         12.767000         38.817000
O         21.085000         14.985000         38.613000
O         22.330000         14.169000         36.730000
C         23.201000         13.197000         36.107000
H         22.686000         12.261000         35.891000
H         24.068000         13.071000         36.755000
C         23.706000         13.683000         34.763000
H         24.117000         12.782000         34.306000
O         22.572000         14.105000         33.983000
C         23.186000         14.930000         33.018000
H         23.438000         14.282000         32.179000
N         22.269000         16.007000         32.466000
C         21.386000         16.736000         33.231000
H         21.284000         16.563000         34.292000
C         20.621000         17.716000         32.667000
H         20.026000         18.361000         33.297000
C         20.694000         17.854000         31.237000
N         19.882000         18.753000         30.677000
H         19.855999         18.767000         29.667000
H         19.159000         19.169000         31.247000
N         21.472000         17.142000         30.512000
C         22.426998         16.331996         31.096000
O         23.332012         15.855029         30.393000
C         24.711000         14.799000         34.855000
H         24.540000         15.490000         35.681000
C         24.467000         15.506000         33.544000
H         24.368000         16.563000         33.791000
H         25.302000         15.286000         32.879000
O         26.078000         14.311000         34.943000
P         27.294695         15.371187         35.287083
O         28.339816         14.554378         35.999334
O         26.742345         16.648152         35.862263
O         27.829690         15.775387         33.791343
C         28.363835         14.741434         32.945232
H         27.564511         14.049446         32.650242
H         29.147962         14.178525         33.468453
C         28.960077         15.375946         31.713862
H         29.388981         14.576055         31.099998
O         27.929310         16.024474         30.918127
C         28.070024         17.428829         30.973367
H         28.237976         17.814247         29.961798
N         26.775448         18.042244         31.388588
C         26.281489         18.268147         32.615500
H         26.792414         17.978608         33.526371
N         25.069286         18.905306         32.618422
C         24.793409         19.083555         31.342563
C         23.641489         19.713047         30.702217
O         22.727475         20.269743         31.290647
N         23.707638         19.629463         29.318015
H         22.928760         20.109293         28.773271
C         24.726790         19.048376         28.619540
N         24.663942         18.999182         27.315103
H         23.826540         19.357093         26.796093
H         25.437152         18.574101         26.811267
N         25.848510         18.486037         29.210459
C         25.824752         18.534594         30.502553
C         30.008519         16.455140         31.988725
H         30.489300         16.315797         32.961832
C         29.217492         17.759402         31.925375
H         28.859401         18.007112         32.925797
H         29.808241         18.598155         31.552924
O         30.998462         16.354383         30.943409
P         32.365628         17.271754         31.018594
O         33.390695         16.514341         30.215192
O         32.623795         17.710872         32.435838
O         31.935256         18.640262         30.218657
C         31.646294         18.544847         28.811475
H         30.638129         18.132726         28.663302
H         32.374273         17.891692         28.313513
C         31.739273         19.909653         28.183653
H         31.699095         19.787104         27.092313
O         30.616679         20.733251         28.603572
C         31.139133         22.038105         28.846718
H         31.272913         22.583686         27.901601
N         30.158485         22.805272         29.595474
C         29.856333         22.784819         30.917708
H         30.371733         22.139856         31.618945
N         28.875778         23.638505         31.259590
C         28.514873         24.212467         30.094629
C         27.521046         25.177598         29.745359
N         26.745858         25.791397         30.614410
H         26.050335         26.467041         30.275883
H         26.832561         25.602156         31.606040
N         27.366631         25.467100         28.421143
C         28.145680         24.894743         27.525661
```

```
H         27.963747         25.158424         26.485215
N         29.156030         23.999133         27.741119
C         29.280539         23.701256         29.012755
C         33.006839         20.714098         28.559239
H         33.786553         20.084873         29.000613
C         32.478531         21.781179         29.512782
H         32.334896         21.361387         30.512959
H         33.127749         22.657237         29.554834
O         33.479742         21.402401         27.389936
P         34.818000         20.869000         26.588000
O         34.661000         19.464000         26.205000
O         36.013000         21.289000         27.308000
O         34.653000         21.797000         25.309000
C         33.640000         21.565000         24.330000
H         32.717000         21.259000         24.822000
H         33.902000         20.776000         23.625000
C         33.345000         22.818000         23.473000
H         32.904000         22.525000         22.520000
O         32.399000         23.658000         24.202000
C         33.072000         24.885000         24.560000
H         32.623000         25.581000         23.852000
N         32.650000         25.181000         25.931000
C         33.256000         24.782000         27.135000
H         34.108000         24.120000         27.082000
N         32.747000         25.297000         28.175000
C         31.694000         26.070000         27.614000
C         30.649000         26.899000         28.140000
N         30.374000         27.107000         29.383000
H         29.550000         27.662000         29.569000
H         30.972000         26.787000         30.131000
N         29.788000         27.536000         27.355000
C         29.855000         27.411000         26.078000
H         29.289000         27.984000         25.358000
N         30.694000         26.561000         25.456000
C         31.630000         26.012000         26.256000
C         34.587999         23.600000         23.122001
H         35.461000         22.961000         23.264000
C         34.524000         24.663000         24.254000
H         35.006000         24.212000         25.121000
H         35.060000         25.591000         24.056000
O         34.587000         24.085000         21.822000
P         22.756000         35.930999         29.114000
O         22.041000         37.123000         28.597000
O         22.217000         35.282000         30.336000
O         22.918000         34.787000         27.935000
C         23.440000         35.066000         26.741000
H         24.076000         35.941000         26.875000
H         22.610000         35.148000         26.039000
C         24.493000         33.994000         26.215000
H         25.084000         34.391000         25.390000
O         25.294000         33.383000         27.206000
C         25.804000         32.177000         26.653000
H         26.623000         32.506000         26.013000
N         26.143000         31.069000         27.662000
C         25.825000         31.117000         28.996000
H         25.081000         31.823000         29.332000
C         26.474000         30.351000         29.916000
C         26.057000         30.349000         31.416000
H         25.219000         31.036000         31.531000
H         25.630000         29.356000         31.554000
H         26.923000         30.483000         32.065000
C         27.470000         29.367000         29.484000
O         28.258000         28.715000         30.179000
N         27.732000         29.393000         28.167999
H         28.539000         28.881000         27.841001
C         27.248000         30.330000         27.289000
O         27.675000         30.242000         26.110000
C         23.606000         32.941000         25.578000
H         22.692000         32.806000         26.157000
C         24.584000         31.730000         25.783000
H         24.085000         30.857000         26.204000
H         24.966000         31.414000         24.813000
O         23.380000         33.126000         24.238000
P         21.979096         32.597416         23.523829
O         21.949066         33.307614         22.198633
O         20.842921         32.664033         24.506405
O         22.292126         31.003340         23.312145
C         23.224875         30.634961         22.273731
H         24.173472         31.172154         22.402932
H         22.808149         30.882370         21.289694
C         23.477584         29.150242         22.333963
H         24.099177         28.882882         21.469713
O         24.205060         28.831685         23.548557
C         23.562324         27.728676         24.187289
H         23.990399         26.782092         23.838478
N         23.872822         27.805050         25.622617
C         23.148237         28.566733         26.478868
H         22.333019         29.138390         26.048633
C         23.399578         28.623015         27.825396
C         22.603478         29.457927         28.751671
H         22.149799         28.837687         29.535197
H         23.255898         30.172231         29.274271
H         21.822822         30.005987         28.221687
C         24.501397         27.840456         28.344969
```

O	24.812272	27.800067	29.545063
N	25.233191	27.141264	27.409575
H	26.032781	26.574987	27.756787
C	25.006775	27.090183	26.062820
O	25.733521	26.475603	25.293680
C	22.211410	28.273216	22.344639
H	21.329517	28.826996	22.010147
C	22.095146	27.826330	23.799809
H	21.566403	28.602857	24.356412
H	21.565453	26.878728	23.910735
O	22.462350	27.153475	21.470421
P	21.214758	26.302490	20.811845
O	21.279503	26.437197	19.313318
O	19.941571	26.607381	21.562646
O	21.725803	24.774461	21.114571
C	21.810683	24.332611	22.478475
H	20.916646	24.625217	23.045880
H	22.700908	24.758415	22.961245
C	21.952118	22.818798	22.441418
H	22.794794	22.540622	21.796735
O	22.225596	22.365807	23.786116
C	21.163799	21.500714	24.217051
H	21.471555	20.462543	24.056719
N	21.004120	21.658521	25.663115
C	20.271724	22.671769	26.201672
H	19.753899	23.330345	25.515417
C	20.187359	22.837826	27.543079
H	19.578734	23.626269	27.967664
C	20.947367	21.965444	28.371791
N	20.894730	22.080023	29.695567
H	21.445730	21.466229	30.289412
H	20.366276	22.833395	30.109860
N	21.733516	21.011107	27.836493
C	21.780204	20.837110	26.502166
O	22.496066	19.954548	25.974222
C	20.670584	22.093914	22.013757
H	20.069602	22.688737	21.317845
C	19.970274	21.841649	23.338603
H	19.467604	22.760702	23.648970
H	19.243889	21.030217	23.282397
O	21.029126	20.817240	21.442874
P	20.918000	20.622000	19.818000
O	21.638000	21.642000	19.083000
O	19.525000	20.336000	19.415000
O	21.783000	19.347000	19.629000
C	21.223000	18.039000	19.642001
H	20.898000	17.693000	18.660000
H	20.385000	18.030000	20.338000
C	22.240000	17.009000	20.062999
H	23.034000	17.158000	19.331000
O	22.704000	17.292000	21.422000
C	21.985000	16.509000	22.397000
H	22.630000	15.759000	22.857000
N	21.280000	17.193000	23.506000
C	20.282000	18.100000	23.346000
H	19.797000	18.394000	22.427000
N	19.704000	18.516000	24.424000
C	20.427000	17.839000	25.417000
C	20.406000	17.904000	26.800000
O	19.771000	18.518000	27.685000
N	21.411001	17.120001	27.411000
H	21.415000	17.047000	28.419000
C	22.301997	16.368994	26.806000
N	23.328018	15.889053	27.405001
H	23.468973	15.827925	28.403999
H	23.975002	15.338002	26.859000
N	22.428000	16.286999	25.458000
C	21.458000	17.025000	24.854000
C	21.726000	15.605001	20.228001
H	20.960000	15.481000	19.463000
C	21.055000	15.538000	21.619000
H	20.003000	15.769000	21.452000
H	21.079000	14.532000	22.039000
O	22.839000	14.723000	20.115000
O	26.505010	21.287468	30.624215
O	24.648328	23.033277	30.578315
O	24.925744	23.219125	25.193466
O	22.813578	24.868057	30.574185
O	27.824347	19.160870	25.406087
C	25.731427	22.280065	27.204781
C	25.665846	22.273868	28.597989
C	23.809208	23.957008	28.570048
C	23.826883	23.960330	27.154588
C	24.696500	23.093549	29.319123
C	24.823968	23.156628	26.415478
C	26.660421	21.476103	26.529130
C	26.566975	21.427096	29.306971
C	27.555798	20.661640	27.229570
C	27.511159	20.639720	28.614701
C	22.885868	24.796518	29.241120
C	22.927469	24.741591	26.440474
C	28.525281	19.790332	26.484408
C	21.995309	25.594124	28.502264
C	22.006221	25.542691	27.122939
H	26.679150	21.500324	25.444737
H	28.190876	20.023284	29.193604
H	22.944745	24.724764	25.355410
H	29.346778	20.414488	26.100239
H	28.954338	19.045104	27.166800
H	21.294390	26.224944	29.040109
H	21.298156	26.143673	26.559949
H	25.786594	21.914169	30.951620
H	23.468234	24.226251	30.946664
H	28.487092	18.767897	24.823573

ENERGIES
FINAL SINGLE POINT ENERGY: -11963.498262192170

==> 0/UVVIS/S1 <==
30

O	1.448374	2.743791	-0.004115
O	-0.990392	2.395698	0.003269
O	-0.220122	-2.974780	-0.010408
O	-3.432147	2.058010	0.008878
O	5.554241	-0.123824	-0.034597
C	0.720487	-0.815192	-0.007673
C	0.505025	0.570918	-0.003915
C	-1.921282	0.229028	0.002798
C	-1.736125	-1.169220	0.000541
C	-0.816358	1.123252	0.001267
C	-0.404350	-1.741955	-0.005837
C	2.027240	-1.292013	-0.012244
C	1.642495	1.448327	-0.006758
C	3.125007	-0.429146	-0.020495
C	2.933181	0.939296	-0.014865
C	-3.254818	0.757967	0.007112
C	-2.859838	-1.994721	0.004096
C	4.492669	-1.034384	-0.036434
C	-4.352357	-0.094028	0.010184
C	-4.140565	-1.459924	0.008602
H	2.189263	-2.363356	-0.010145
H	3.767203	1.628740	-0.021628
H	-2.720449	-3.067782	0.003127
H	4.570639	-1.736690	0.805165
H	4.595211	-1.638107	-0.944392
H	-5.348382	0.330580	0.013336
H	-4.993359	-2.129067	0.010539
H	-2.500374	2.473995	0.006195
H	5.599865	0.307018	0.827555
H	0.433934	2.881739	-0.001170

ENERGIES
FINAL SINGLE POINT ENERGY: -952.291740185968
FINAL GIBBS FREE ENERGY: -952.11651708

==> 0/UVVIS/T1 <==
30

O	1.483378	2.716780	-0.006499
O	-0.950933	2.423333	0.000954
O	-0.204008	-2.949461	-0.006173
O	-3.503260	2.085477	0.008241
O	5.584196	-0.153470	-0.040907
C	0.724680	-0.795679	-0.007350
C	0.515037	0.573773	-0.005180
C	-1.918006	0.268256	0.002370
C	-1.727239	-1.142095	0.000662
C	-0.798330	1.111889	-0.000597
C	-0.395517	-1.717130	-0.004309
C	2.051995	-1.293448	-0.012666
C	1.662941	1.466831	-0.008378
C	3.157262	-0.454180	-0.020298
C	2.967162	0.910383	-0.015507
C	-3.246986	0.779093	0.006801
C	-2.834189	-1.981232	0.003680
C	4.525387	-1.069904	-0.034754
C	-4.322312	-0.088582	0.009842
C	-4.111984	-1.458074	0.008303
H	2.191729	-2.367559	-0.011477
H	3.804178	1.596947	-0.019899
H	-2.680557	-3.051831	0.002381
H	4.605619	-1.763020	0.811725
H	4.624876	-1.672727	-0.942133
H	-5.323167	0.325641	0.013324
H	-4.966233	-2.124983	0.010708
H	-2.658430	2.579776	0.005704
H	5.652941	0.253341	0.831131
H	0.003683	2.816024	-0.001713

ENERGIES
FINAL SINGLE POINT ENERGY: -952.315276308481
FINAL GIBBS FREE ENERGY: -952.13866686

==> 0/UVVIS/T1/AEA <==
30

O	1.436722	2.821482	-0.274153

```
O         -1.010216          2.427722         -0.170235
O         -0.156239         -2.940974          0.107287
O         -3.459684          2.058417         -0.048614
O          5.276589         -0.506516          0.984326
C          0.749247         -0.767704         -0.047741
C          0.515445          0.625626         -0.116471
C         -1.905563          0.247224         -0.003998
C         -1.693758         -1.150872          0.070297
C         -0.807309          1.149275         -0.100282
C         -0.353330         -1.701204          0.046521
C          2.063410         -1.241830         -0.067206
C          1.628247          1.500937         -0.205100
C          3.133236         -0.378658         -0.157474
C          2.911066          0.998683         -0.227525
C         -3.232706          0.744083          0.020794
C         -2.795104         -2.004686          0.167403
C          4.534107         -0.903065         -0.161757
C         -4.301553         -0.125697          0.117979
C         -4.073814         -1.495987          0.190810
H          2.236395         -2.309013         -0.010365
H          3.743385          1.689705         -0.302099
H         -2.628358         -3.072007          0.224643
H          4.519156         -1.994214         -0.249232
H          5.084799         -0.504476         -1.017347
H         -5.307364          0.277414          0.136413
H         -4.920523         -2.169173          0.267248
H         -2.542275          2.479074         -0.111557
H          4.836891         -0.870394          1.762487
H          0.433012          2.940998         -0.247063

# ENERGIES
FINAL SINGLE POINT ENERGY: -952.519914035136
FINAL GIBBS FREE ENERGY: -952.34294721

==> 0/UVVIS/T1/AIP <==
30

O          1.447404          2.704710          0.000035
O         -0.924841          2.376733          0.002288
O         -0.218106         -2.944054         -0.011273
O         -3.496734          2.070739          0.007578
O          5.585391         -0.143580         -0.061915
C          0.746658         -0.805369         -0.009747
C          0.527994          0.554889         -0.004162
C         -1.926341          0.254222          0.001069
C         -1.748704         -1.149051         -0.001541
C         -0.794945          1.098284         -0.000161
C         -0.399175         -1.741491         -0.007768
C          2.050650         -1.289603         -0.015891
C          1.655744          1.463098         -0.003732
C          3.166243         -0.428204         -0.018580
C          2.973740          0.922263         -0.009317
C         -3.241543          0.783537          0.005807
C         -2.841508         -1.977348          0.001762
C          4.525576         -1.049693         -0.029470
C         -4.336655         -0.088157          0.009150
C         -4.133842         -1.440276          0.007391
H          2.204850         -2.362616         -0.018182
H          3.805123          1.615910         -0.009789
H         -2.697974         -3.049511          0.000078
H          4.597269         -1.728720          0.830610
H          4.604593         -1.677028         -0.923362
H         -5.332436          0.337287          0.013141
H         -4.986448         -2.108632          0.010265
H         -2.661915          2.583479          0.005398
H          5.680075          0.266443          0.806705
H          0.083770          2.775908          0.001601

# ENERGIES
FINAL SINGLE POINT ENERGY: -952.150127673032
FINAL GIBBS FREE ENERGY: -951.97365112

==> 0/UVVIS/T2 <==
30

O          1.480199          2.754915         -0.006012
O         -0.997246          2.409669          0.000988
O         -0.231699         -2.947867         -0.000691
O         -3.466190          2.049688          0.006671
O          5.550437         -0.122170         -0.028750
C          0.712615         -0.792191         -0.005837
C          0.503518          0.592927         -0.004520
C         -1.926708          0.245371          0.003044
C         -1.735600         -1.142584          0.002469
C         -0.814499          1.140942         -0.000001
C         -0.407572         -1.703075         -0.001612
C          2.027815         -1.295001         -0.011311
C          1.637629          1.448247         -0.008747
C          3.122775         -0.448864         -0.021089
C          2.927590          0.922775         -0.017749
C         -3.252940          0.753730          0.006354
C         -2.863771         -2.001967          0.005597
C          4.498361         -1.048368         -0.040348
C         -4.347260         -0.115185          0.009242
C         -4.137745         -1.487352          0.008850
H          2.171057         -2.368212         -0.008974
H          3.764991          1.608583         -0.024460
H         -2.699447         -3.070735          0.005083
H          4.585343         -1.754270          0.794720
H          4.609476         -1.634876         -0.957020
H         -5.344576          0.307079          0.011648
H         -4.990250         -2.155691          0.011055
H         -2.547824          2.482398          0.004481
H          5.604627          0.280108          0.846285
H          0.480807          2.916145         -0.001382

# ENERGIES
FINAL SINGLE POINT ENERGY: -952.304181525391
FINAL GIBBS FREE ENERGY: -952.12491022

==> 0/UVVIS/T3 <==
30

O          1.474088          2.766334         -0.007984
O         -0.996231          2.417692          0.003101
O         -0.225608         -2.924005          0.003327
O         -3.463545          2.062999          0.006109
O          5.531681         -0.126965         -0.026300
C          0.721823         -0.790321         -0.005459
C          0.499566          0.600471         -0.003792
C         -1.922942          0.251716          0.004006
C         -1.737734         -1.145101          0.003763
C         -0.814401          1.151177          0.001983
C         -0.407952         -1.669949         -0.000146
C          2.013848         -1.311306         -0.011033
C          1.628540          1.449078         -0.009946
C          3.101407         -0.457830         -0.020912
C          2.908001          0.915784         -0.018824
C         -3.245026          0.754614          0.005965
C         -2.833722         -2.013663          0.006683
C          4.481181         -1.054393         -0.041802
C         -4.321889         -0.118600          0.008237
C         -4.105810         -1.489205          0.008538
H          2.157137         -2.385446         -0.009915
H          3.751905          1.593576         -0.027222
H         -2.670507         -3.083805          0.006487
H          4.569051         -1.761954          0.791438
H          4.593733         -1.636379         -0.960964
H         -5.325175          0.289729          0.009706
H         -4.958836         -2.158239          0.009792
H         -2.555579          2.497307          0.004559
H          5.586090          0.270356          0.850937
H          0.480818          2.930498         -0.002345

# ENERGIES
FINAL SINGLE POINT ENERGY: -952.293138922050
FINAL GIBBS FREE ENERGY: -952.11625594

==> 0/UVVIS/T4 <==
30

O          1.472982          2.794520         -0.134952
O         -0.978029          2.420556         -0.098645
O         -0.201565         -2.973948          0.048555
O         -3.450350          2.072538         -0.028548
O          5.405791         -0.201983          0.587660
C          0.739072         -0.821437         -0.018331
C          0.519904          0.610126         -0.057926
C         -1.904402          0.253683         -0.005765
C         -1.708127         -1.160744          0.038126
C         -0.801660          1.150323         -0.060278
C         -0.379666         -1.740790          0.028044
C          2.032177         -1.319334         -0.028102
C          1.634918          1.480148         -0.101457
C          3.108576         -0.464114         -0.089714
C          2.905520          0.951770         -0.126896
C         -3.222487          0.764142          0.011437
C         -2.818128         -2.010837          0.092795
C          4.497241         -0.977515         -0.144722
C         -4.297139         -0.105369          0.069819
C         -4.085045         -1.489431          0.110678
H          2.189488         -2.388387          0.014451
H          3.757353          1.616103         -0.193945
H         -2.656916         -3.079167          0.125754
H          4.519495         -2.028117          0.172845
H          4.839174         -0.965477         -1.188756
H         -5.300744          0.302436          0.081748
H         -4.943482         -2.149137          0.158651
H         -2.545580          2.506621         -0.065731
H          5.111369         -0.165701          1.507221
H          0.474174          2.942690         -0.126032

# ENERGIES
FINAL SINGLE POINT ENERGY: -952.286924641888
FINAL GIBBS FREE ENERGY: -952.10638681
```
